# The anterior cingulate cortex controls the hyperactivity in subthalamic neurons in male mice with comorbid chronic pain and depression

**DOI:** 10.1371/journal.pbio.3002518

**Published:** 2024-02-22

**Authors:** Ying-Di Wang, Shu-Ting Bao, Yuan Gao, Jin Chen, Tao Jia, Cui Yin, Jun-Li Cao, Cheng Xiao, Chunyi Zhou

**Affiliations:** 1 Jiangsu Province Key Laboratory of Anesthesiology, School of Anesthesiology, Xuzhou Medical University, Xuzhou, Jiangsu, China; 2 Jiangsu Province Key Laboratory of Anesthesia and Analgesia Application Technology, Xuzhou Medical University, Xuzhou, Jiangsu, China; 3 NMPA Key Laboratory for Research and Evaluation of Narcotic and Psychotropic Drugs, School of Anesthesiology, Xuzhou Medical University, Xuzhou, Jiangsu, China; 4 Wuxi Ninth People’s Hospital Affiliated to Soochow University, Wuxi, Jiangsu, China; 5 Department of Anesthesiology, The Affiliated Hospital of Xuzhou Medical University, Xuzhou Medical University, Xuzhou, Jiangsu, China; Columbia University Irving Medical Center, UNITED STATES

## Abstract

Neurons in the subthalamic nucleus (STN) become hyperactive following nerve injury and promote pain-related responses in mice. Considering that the anterior cingulate cortex (ACC) is involved in pain and emotion processing and projects to the STN, we hypothesize that ACC neurons may contribute to hyperactivity in STN neurons in chronic pain. In the present study, we showed that ACC neurons enhanced activity in response to noxious stimuli and to alterations in emotional states and became hyperactive in chronic pain state established by spared nerve injury of the sciatic nerve (SNI) in mice. In naïve mice, STN neurons were activated by noxious stimuli, but not by alterations in emotional states. Pain responses in STN neurons were attenuated in both naïve and SNI mice when ACC neurons were inhibited. Furthermore, optogenetic activation of the ACC-STN pathway induced bilateral hyperalgesia and depression-like behaviors in naive mice; conversely, inhibition of this pathway is sufficient to attenuate hyperalgesia and depression-like behaviors in SNI mice and naïve mice subjected to stimulation of STN neurons. Finally, mitigation of pain-like and depression-like behaviors in SNI mice by inhibition of the ACC-STN projection was eliminated by activation of STN neurons. Our results demonstrate that hyperactivity in the ACC-STN pathway may be an important pathophysiology in comorbid chronic pain and depression. Thus, the ACC-STN pathway may be an intervention target for the treatment of the comorbid chronic pain and depression.

## Abstract 2

Neurons in the subthalamic nucleus (STN) are hyperactive in chronic pain and depression but how this activity is regulated is unclear. This study shows that neurons projecting into the STN from the anterior cingulate cortex, which integrates pain signals with negative emotions, regulate the activity of STN neurons.

## Introduction

Chronic pain affects approximately 15% of the population and is commonly associated with psychological maladaptations, including depression [1,2]. The presence of depression prolongs and exacerbates pain [2,3]. The interplay between pain and depression creates a vicious cycle that poses a significant challenge to effective treatment for comorbid chronic pain and depression. Fortunately, many neuroplasticity changes have been shared by chronic pain and depression [4–11]. Identifying neural circuit pathways implicated in chronic pain and depression will enrich the repertoire of targets used to develop effective therapies for the comorbid chronic pain and depression.

The subthalamic nucleus (STN), an innate glutamatergic nucleus in the basal ganglia, has been extensively investigated in both patients with Parkinson’s disease (PD) and rodent PD models. Evidence demonstrates that deep brain stimulation in the STN relieves pain in PD patients [12,13]. Alterations in neuronal activity of the STN also correlate with depression in PD patients [14,15]. Moreover, STN neurons readily respond to painful and emotional stimuli [16–18]. Our group showed that activation of STN neurons is sufficient to trigger pain-like behavior and emotional dysfunction in naive mice; inhibition of the hyperactive STN neurons and some of their projections in chronic pain mouse models alleviates pain- and depression-like behaviors [7,17,19,20]. These results implicate the STN in the pathophysiology of comorbid chronic pain and depression. However, the mechanisms underlying dysfunctions of STN neurons and their related circuits remain poorly understood.

Cortical regions, including the anterior cingulate cortex (ACC), play important roles in the high-level integration of pain signals with negative emotions. Because ACC neurons carry value-related information such as rewards, fear, or pain [21–24], it plays a key role in regulating emotional states associated with pain [25]. In humans, neuroimaging studies have consistently found that patients with chronic pain and depression exhibit hyperactivity in the ACC [26,27]. In terms of neural circuits, the ACC is among the cortical inputs to the STN forming the hyperdirect pathway in the cortico-basal ganglia-thalamo-cortical loop [28–31]. However, it remains unknown whether the ACC plays a role in the enhancement of STN neuron activity in chronic pain and whether the ACC and STN coordinate to control the manifestation of pain- and depression-like behaviors in chronic pain.

In the present study, we hypothesized that the ACC controls pain and emotional responses in STN neurons and the dysfunction of the ACC-STN pathway is an important pathophysiology contributing to the comorbid chronic pain and depression. We observed that ACC neurons were hyperactive in mice subjected to the spared nerve injury (SNI), a chronic pain model; inhibition of STN-projecting ACC neurons attenuated pain-like and aversion-like responses in STN neurons in SNI mice; optogenetic activation of the ACC-STN pathway is sufficient to trigger hyperalgesia and depression-like behaviors in naive mice; optogenetic inhibition of the ACC-STN pathway ameliorated hyperalgesia and depression-like behavior in SNI mice, which was annihilated by chemogenetic activation of STN neurons. Overall, this study demonstrated a significant role of the ACC-STN pathway in the pathophysiology of comorbid chronic pain and depression.

## Results

### STN-projecting ACC neurons are hyperactive in neuropathic pain

**Fig 1:**
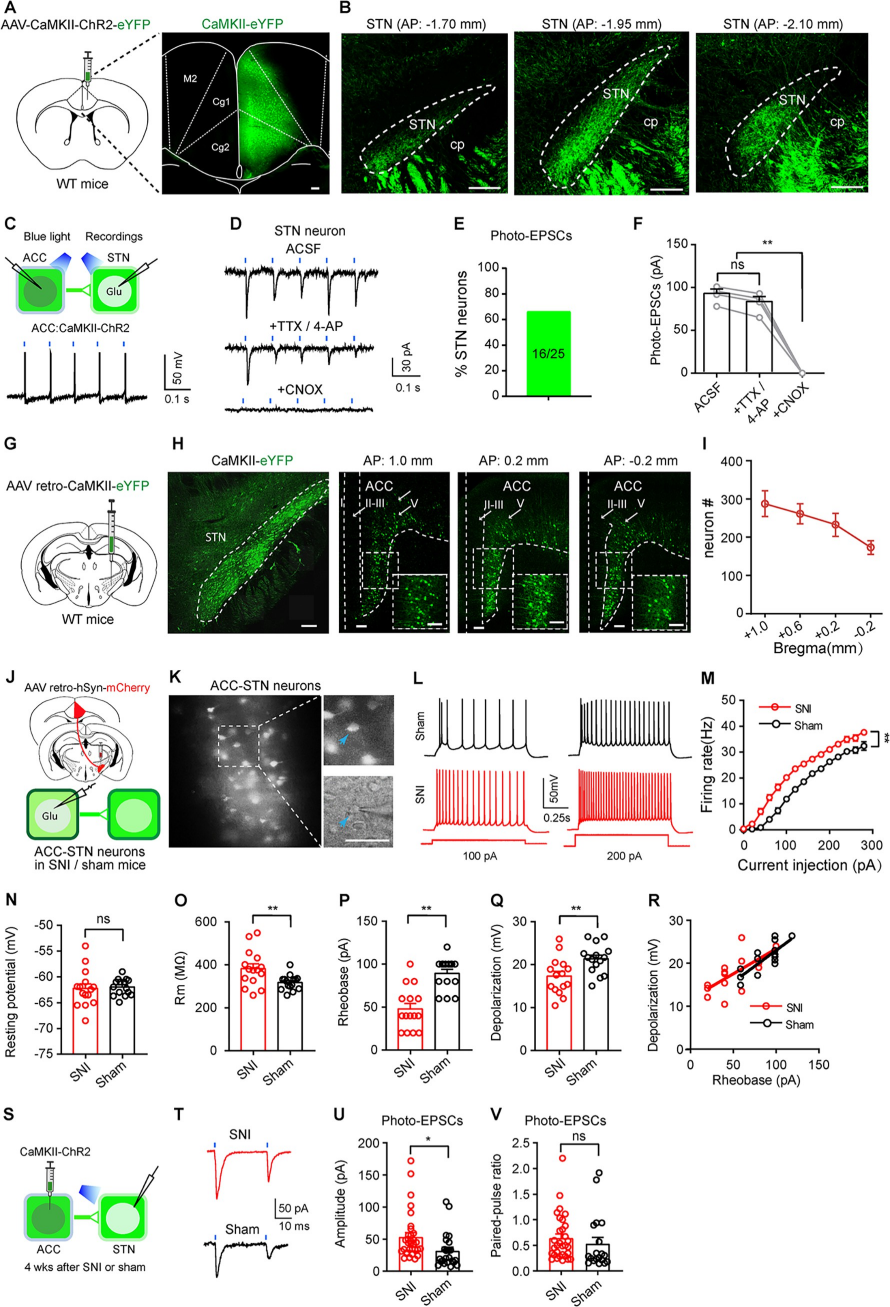
STN-projecting ACC neurons are hyperactive in chronic pain. (**A and B**) Viral vector was injected in the ACC to label ACC neurons **(A)** and enable visualization of their projections to the STN **(B)**. **(C)** Whole-cell patch-clamp recordings were performed to record firing in ACC neurons evoked by 5 ms blue light stimulation (50 ms interval, 2 mW) (upper panel, schematic diagram; lower panel, a typical trace). (**D–F**) Blue light (5 ms, 10 Hz, 2 mW)-evoked excitatory postsynaptic currents (photo-EPSCs) in STN neurons were not blocked by perfusion of 1 μM TTX and 0.3 mM 4-AP, but were blocked by 20 μM CNQX. **(D)** Traces. **(E)** Percentage of STN neurons (25 neurons from 4 mice) responding to photostimulation of the ACC-STN projection. **(F)** Summary of amplitude of photo-EPSCs in different conditions (F_(2, 9)_ = 125.5, *P* < 0.0001, *n* = 8 neurons recorded from 4 mice). (**G and H**) Schematic diagram **(G)**, image **(H)**, and summary **(I)** showing that virus injected into the STN retrogradely labeled layer V neurons in the ACC in coronal sections (*n* = 5 mice). (**J**) Schematic diagram for retrograde labeling of STN-projecting ACC neurons and whole-cell patch-clamp recording of STN-projecting ACC neurons 4 weeks after SNI or sham surgery. (**K**) Left: A representative image of mCherry-labeled STN-projecting ACC neurons in the layer V of the ACC from an SNI mouse 4 weeks after SNI surgery. Right: higher magnification fluorescent image (upper right) and IR-DIC image (lower right). Arrow head points to a patched mCherry-labeled ACC neuron. (**L and M)** Representative traces **(L)** and summary **(M)** of evoked firing recorded from mCherry-labeled ACC neurons 4 weeks after sham (black) or SNI (red) surgery (Group: F_(1, 806)_ = 241.6, *P* < 0.0001; *n* = 37 neurons in SNI, *n* = 29 neurons in sham, from 4 mice in each group). (**N–Q**) Quantification of resting membrane potential (N, t = 0.28, *P* = 0.78, *n* = 15 neurons in each group), membrane resistance (O, Rm, t = 2.77, *P* = 0.0098, *n* = 15 neurons in each group), rheobase (P, t = 5.19, *P* < 0.0001, *n* = 15 neurons in each group), and depolarization threshold (Q, t = 2.86, *P* = 0.008, *n* = 15 neurons in each group) of STN-projecting ACC neurons from SNI and sham mice. (**R**) Scatter plot and linear relationship between depolarization voltage and rheobase in SNI and sham mice. SNI: r = 0.68, *P* = 0.0058; sham: r = 0.80, *P* = 0.0004; *n* = 15 neurons in each group. *P* = 0.51, SNI vs. sham mice. **(S)** Diagram showing AAV-CaMKII-ChR2-eYFP injection into the ACC and patch-clamp recording from STN neurons of SNI and sham mice. **(T)** Photo-EPSCs recorded from STN neurons in SNI (red) and sham (black) mice. **(U)** Amplitude of photo-EPSCs in STN neurons from SNI and sham mice (t = 2.45, *P* = 0.018, *n* = 32 neurons in SNI, *n* = 24 neurons in sham, from 4 mice in each group). **(V)** Paired-pulse ratio of photo-EPSCs in STN neurons from sham and SNI mice (t = 0.78, *P* = 0.44, *n* = 31 neurons in SNI, *n* = 19 neurons in sham, from 4 mice in each group). * *P* < 0.05, ** *P* < 0.01; one-way repeated measures ANOVA with Tukey’s post hoc analysis for **(E)**; two-way repeated measures ANOVA with Tukey’s post hoc analysis for **(M)**; two-tailed unpaired *t* test for **(N–Q, U, V)**. A z-test on Fisher z-transformed correlation coefficients was used in **(R)**. Scale bars: 100 μm. Source data can be found in the first worksheet of S1 Data. ACC, anterior cingulate cortex; EPSC, excitatory postsynaptic current; SNI, spared nerve injury; STN, subthalamic nucleus.

To provide synaptic evidence for the projection from the ACC to the STN (ACC-STN projection), we injected AAV-CaMKII-ChR2-eYFP into the ACC of wild-type (WT) mice (Fig 1A). Four weeks later, we observed a great many of eYFP-labeled neurons in the ACC and eYFP-labeled axonal fibers in the STN (Fig 1A and 1B). Whole-cell patch-clamp recording from acute brain slices showed that eYFP-labeled ACC neurons displayed time-locked firing in response to blue light stimulation (473 nm, 20 Hz, 5 ms; Fig 1C); in 64% (16 out of 25 neurons from 4 mice) of STN neurons among eYFP-labeled fibers, 5 blue light pulses (473 nm, 5 ms, 20 Hz) evoked excitatory postsynaptic currents (photo-EPSCs) (Fig 1D and 1E). These photo-EPSCs were not blocked by the mixture of tetrodotoxin (TTX; 1 μM), a voltage-dependent sodium channel blocker, and 300 μM 4-aminopyridine (4-AP), a blocker of voltage-gated potassium channels, but were abolished by subsequent application of CNQX (20 μM), an antagonist for glutamate receptors (Fig 1D and 1F). The amplitude and latency for light-evoked EPSCs were 91.57 ± 4.95 pA (*n* = 4 mice) (Fig 1E) and 5.5 ± 0.3 ms (*n* = 4 mice), respectively. These data demonstrate a valid protocol to manipulate the monosynaptic glutamatergic ACC-STN projection.

To visualize the location of STN-projecting ACC neurons (ACC-STN neurons), we injected a retrograde AAV vector (AAV retro-CaMKII- eYFP) into the STN of WT mice (Fig 1G). After 3 weeks’ recovery, we observed numerous eYFP-labeled neurons along the anterior-posterior axis in the ACC, more specifically, in layer V (Fig 1H and 1I). The results show that ACC-STN neurons distribute throughout the ACC preferentially in layer V.

We then established SNI neuropathic pain mouse models and examined whether the excitability of ACC-STN neurons is modified in SNI mice. The mice subjected to SNI surgery on the right side exhibited long-lasting mechanical and thermal hypersensitivity since the third day after surgery and developed depression-like behaviors 5 weeks after surgery (S1A–S1F Fig). Our histological data showed that c-Fos-positive neurons in the ACC were significantly increased 4 weeks after surgery in SNI mice (S1G and S1H Fig). These data support that ACC neurons are hyperactive in neuropathic pain, consistent with a previous study [32].

We next examined whether ACC-STN neurons were among the hyperactive ACC neurons in SNI mice. To answer this question, we injected AAV retro-hSyn-mCherry-Cre into the STN to label ACC-STN neurons, then we performed whole-cell patch-clamp recording from the labeled ACC-STN neurons 4 weeks after SNI or sham operation (Fig 1J and 1K). The excitability of ACC-STN neurons in SNI mice was enhanced significantly compared with that in sham mice (Fig 1L–1R). Specifically, ACC-STN neurons in SNI mice responded to depolarizing current injections with higher firing rates than those in sham mice (Fig 1L and 1M). In addition, ACC-STN neurons displayed higher membrane resistance, lower rheobase, depolarization threshold to evoke action potentials, though resting membrane potentials, the correlation between rheobase and membrane depolarization did not differ significantly between SNI and sham mice (Fig 1N–1R). We then performed patch-clamp recording from STN neurons in mice subjected to AAV-CaMKII-ChR2-eYFP injection into the ACC (Fig 1S and 1T). We observed bigger amplitude, but similar paired pulse ratio of photo-EPSCs in STN neurons in SNI mice, relative to sham mice (Fig 1U and 1V).

These data suggest that chronic pain not only enhances the excitability of ACC-STN neurons, but also synaptic strength of the ACC-STN projection.

### ACC and STN neurons respond similarly to pain-like and aversive stimulation but differently to alterations in emotional states

The ACC receives highly diverse somatosensory inputs [21,33]. Accumulating evidence suggests that the ACC and STN may have common roles in pain processing in both somatosensory and emotional aspects [5,7,17,19,34,35]. However, comparison of the role of the ACC and STN in these aspects in the same study has been lacking, and it remains unknown whether and how the ACC-STN pathway regulates pain responses in STN neurons. We conducted several experiments to address these issues.

**Fig 2:**
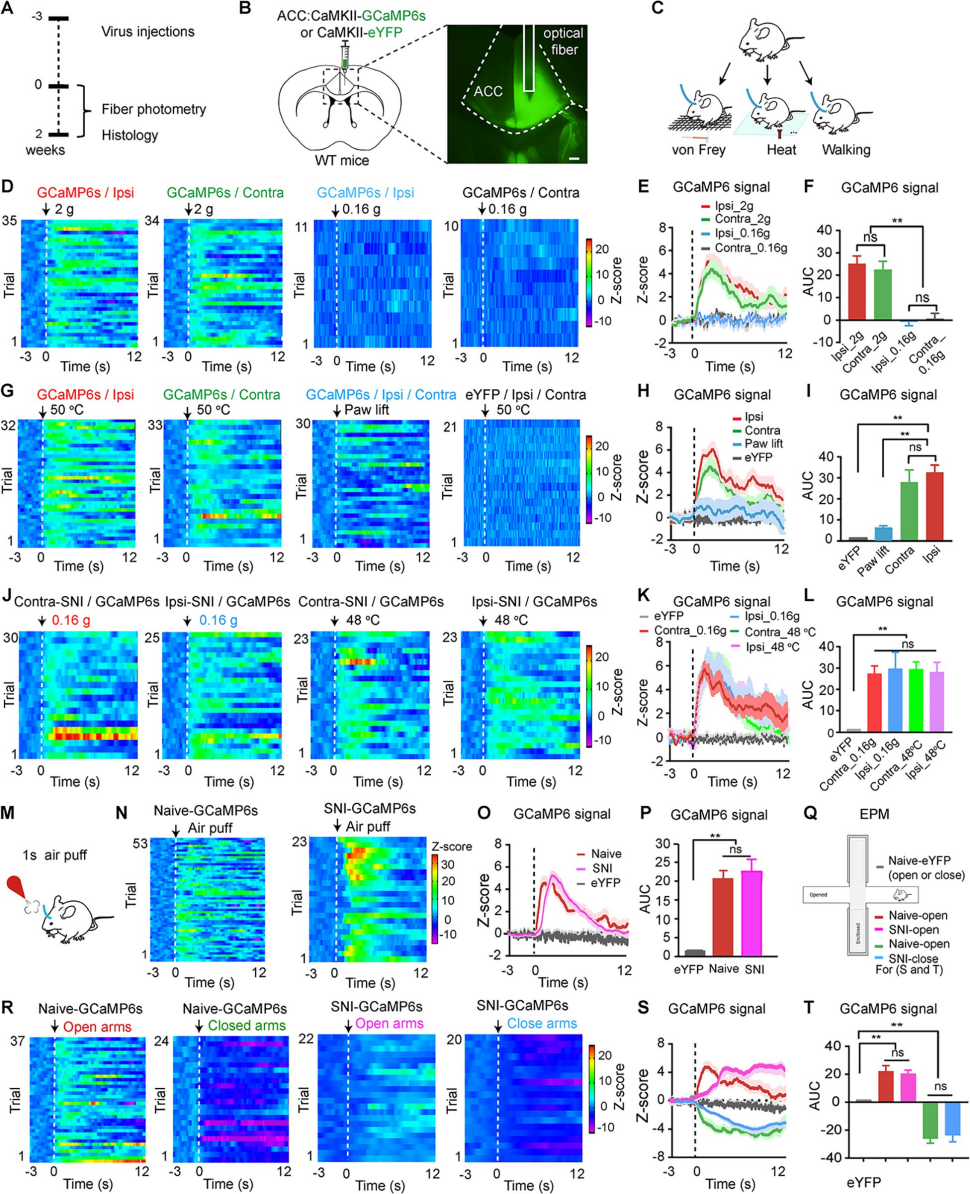
ACC neurons respond to pain-like stimuli and alterations in emotional states. **(A–C)** Schematic diagram **(A**, **C)** and a representative image **(B)** of virus injection into the ACC for fiber photometry recording of GCaMP6s- or eYFP-labeled ACC neurons in response to von Frey filament and heat stimulation on the hind paws or during paw lifting in freely moving mice, and 2 g but not 0.16 g von Frey filament induced paw withdrawal behavior in naïve mice. (**D–I**) Heat maps (**D, G**), averaged normalized traces **(E, H),** and summary (**F, I)** of changes in GCaMP6s and eYFP signals in the ACC in response to von Frey filament stimulation (**F**, F_(3, 86)_ = 8.57, *P* < 0.0001) or thermal stimulation (**I**, F_(3, 118)_ = 16.23, *P* < 0.0001) on hind paws or in response to random paw lifting in naïve mice. *n* = 5 mice in each group. (**J–L**) Heat maps (**J**), averaged normalized traces **(K),** and summary (**L**) of changes in GCaMP6s signals in response to 0.16 g von Frey filament or 48°C thermal stimulation on the hind paw on the injured side in SNI mice that spared nerve injury was performed on the side either contralateral or ipsilateral to GCaMP6s side. (**L**) F_(4, 108)_ = 7.75, *P* < 0.0001. *n* = 5 mice in each group. (**M–P**) Schematic diagram (**M**), heat maps (**N**), averaged normalized traces **(O),** and summary (**P,** F_(2, 110)_ *=* 38.77, *P* < 0.0001) of changes in GCaMP6s and eYFP signals in naïve or SNI mice in response to 1 s air puff onto the face. (**Q–T**) Schematic diagram (**Q**), heat maps (**R**), averaged normalized traces **(S),** and summary (**T,** F_(4, 118)_ = 42.97, *P* < 0.0001) of changes in GCaMP6s and eYFP signal in naïve or SNI mice during exploration of the open or closed arms in the EPM. **(Q)** Includes color coding for **(S)** and **(T)**. ** *P* < 0.01; one-way ANOVA with Tukey’s post hoc analysis for **(F, I, L, P, and T).** *n* = 5 mice in each group. Dashed lines in **(D, E, G, H, J, K, N, O, and S)** indicate the onset of the stimulus. Dashed lines in **(R)** indicate entering the open or closed arms in the EPM. Scale bars: 100 μm. AUC, area under the curve; Contra: Contralateral; Ipsi: Ipsilatera. Source data can be found in the second and third worksheets of S1 Data. ACC, anterior cingulate cortex; EPM, elevated plus maze; SNI, spared nerve injury.

We first monitored the responses of ACC neurons to pain-like stimulation and to events causing changes in emotional states using in vivo fiber photometry. To fulfill this, we transfected AAV-CaMKII-GCaMP6s into excitatory neurons across all layers of the right ACC and implanted an optical fiber above the injection site (Fig 2A and 2B). Three weeks later, we recorded GCaMP6 signal from ACC neurons in freely moving mice upon mechanical and thermal stimuli (Fig 2C). The GCaMP6s signal exhibited a significant increase when the mice showed pain-like responses to a mechanical stimulus with a von Frey filament (with a fiber force of 2 g) or a thermal stimulation (with a 50°C metal block) on both the ipsilateral and contralateral hind paws (Fig 2D–2I). But the GCaMP6s signal in the ACC remained unchanged when the mice were subjected to hind paw stimulation by a sub-threshold von Frey filament (a force of 0.16 g) and while the mice lifted their paws during free exploration in the test chamber (Fig 2D–2I). SNI mice exhibited a comparable enhancement in GCaMP6s signal when subjected to the von Frey filament stimulation corresponding to a supra-threshold mechanical stimulation (0.16 g) or a 48°C thermal stimulation on the hind paws (Fig 2J–2L). Control mice expressing eYFP did not show any fluorescence change in response to von Frey filament or thermal stimulation (Figs 2G–2I and S2A–S2D). These results confirm that hyperactivity of ACC neurons is associated with pain-like hypersensitivity.

To investigate the effect of stimuli related to negative emotional states on GCaMP6s signal in the ACC, we conducted the following experiments. First, we applied 1 s air puff (an aversive stimulation) onto the faces of the mice (Fig 2M) and observed a significant increase in GCaMP6s signal in the ACC (Fig 2N–2P). When the mice explored in an elevated plus maze (EPM) (Fig 2Q), we observed an increase in GCaMP6s signal upon their entrance into the open arms, but a subsequent decrease in the GCaMP6s signal upon their retreat into the closed arms (Fig 2R–2T). In separate trials, changes of GCaMP6s signal in the ACC were observed in SNI mice when they were subjected to air puff onto the face, and explored the open and closed arms in the EPM with similar amplitude as naïve mice, and such effects were not observed in eYFP mice (Figs 2N–2P, 2R, 2T, and S2E–S2J). These results from eYFP mice indicate that changes in GCaMP6s signal were unlikely caused by motion and artifacts.

In summary, the data from GCaMP6 mice suggest that ACC neurons in both naïve and SNI mice detect pain-like stimulation and alterations in emotional states.

**Fig 3:**
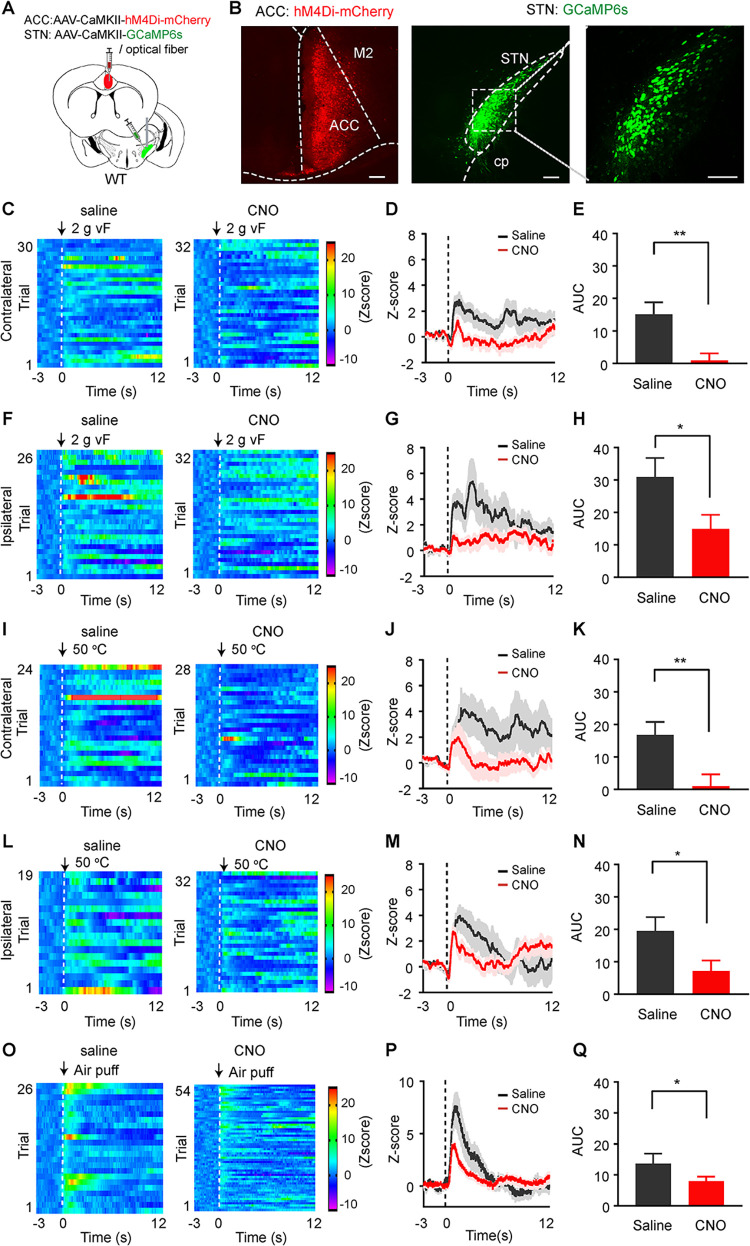
Chemogenetic inhibition of ACC neurons attenuates the response of STN neurons to pain-like and aversive stimuli. **(A)** Experimental strategy for recording of GCaMP6s signal in STN neurons with or without chemogenetic inhibition of ACC neurons. **(B)** Example images of hM4Di and GCaMP6s expression in the injection sites. **(C–N)** Heat maps (**C, F, I,** and **L**), average traces (**D**, **G**, **J**, and **M**), and summary (**E, H, K,** and **N**) of GCaMP6s signal in the STN of mice receiving von Frey filament (2 g) or thermal stimulation on hind paws with intraperitoneal (i.p.) injection of saline or CNO (3 mg/kg). **(E)** *t* = 3.54, *P* = 0.0008. **(H)** *t* = 2.18, *P* = 0.039. **(K)** *t* = 2.91, *P* = 0.0062. **(N)** *t* = 2.29, *P* = 0.03. (**O–Q**) Heat maps (**O**), average traces (**P**), and summary (**Q**, t = 2.21, *P* = 0.029) of GCaMP6s signal in the STN of mice exposed to 1 s air puff onto the face after intraperitoneal injection of saline or CNO. CNO or the same volume of saline was applied 45 min prior to initiation of GCaMP6s signal recording. Dashed lines in (**C**, **D**, **F**, **G**, **I**, **J**, **L**, **M**, **O**, **P**) indicate the onset of the stimulation. * *P* < 0.05, ** *P* < 0.01; two-tailed unpaired *t* test for panels (**E**, **H**, **K**, **N**, and **Q**, *n* = 5 mice.). Scale bars: 100 μm. Source data can be found in the fourth worksheet of S1 Data. ACC, anterior cingulate cortex; CNO, clozapine-N-oxide; STN, subthalamic nucleus.

Our previous studies showed that STN neurons can also be activated by pain-like stimulation and alternation in emotional states [17,20], we next asked whether ACC neurons contribute to pain-like and aversive responses in STN neurons. To solve this puzzle, we injected AAV-CaMKII-hM4Di-mCherry or AAV-CaMKII-mCherry into the ACC and AAV-CaMKII-GCaMP6 into the STN and implanted an optical fiber in the STN (Figs 3A, 3B, and S3A). In this configuration, ACC neurons can be inhibited by clozapine-N-oxide (CNO), the ligand of hM4Di. After ACC neurons were inhibited by intraperitoneally administered CNO (3 mg/kg), the increase in GCaMP6 signal in the STN evoked by von Frey filament, thermal stimulation and air puff were significantly attenuated (Fig 3C–3Q). In control mice that mCherry was virally transfected into ACC neurons, intraperitoneal administration of CNO did not alter GCaMP6s responses of STN neurons to mechanical and thermal stimulation (S3B–S3M Fig). Furthermore, SNI mice exhibited similar GCaMP6s responses of STN neurons to mechanical and thermal stimulation as naïve mice (S1 Table), which were attenuated by chemogenetic inhibition of ACC neurons (S4A–S4X Fig). These results indicate that ACC neurons regulate pain signals in STN neurons in both physiological and neuropathic pain states.

Considering that ACC neurons respond to emotional stimulation [23,36], we investigated whether the activity of STN neurons changes in response to emotional stimulation, and these responses are modulated by ACC neurons in naïve and SNI mice. Similar to the response of ACC neurons to air puff, we found that transient air puff reliably elicited an increase in GCaMP6s signal in STN neurons of both naïve and SNI mice and chemogenetic inhibition of ACC neurons attenuated the responses (Figs 3O–3Q and S4Y–S4Z2 and S2 Table). Air puff-evoked GCaMP6s responses were not affected by CNO in control mice with mCherry-expressing ACC neurons (S3N–S3P Fig). Unlike ACC neurons, STN neurons in naïve mice did not exhibit similar changes in GCaMP6s signal during exploration in the EPM (S5A–S5L Fig). Additionally, intraperitoneal administration of CNO to the mice expressing hM4Di or mCherry in the ACC had no discernible impact on GCaMP6s signal in the STN during EPM exploration (S5A–S5L Fig). In contrast, we observed significant increase or decrease in GCaMP6s signal in the STN of SNI mice upon their entry into the open arms and into the closed arms in the EPM (S2 Table); chemogenetic inhibition of ACC neurons by CNO did not attenuate the alterations of GCaMP6s signal when the mice entered into the open or closed arms (S5M–S5O Fig). These results indicate that STN neurons in neuropathic pain state respond to aversive stimulation and changes in emotional state.

Taken together, these data suggest that ACC neurons influence the processing of pain signals and emotion in the STN. In the following paragraphs, we determined the behavioral consequences of modulating the ACC-STN pathway.

### Activating ACC-STN neurons induces hyperalgesia and depression-like behaviors in naïve mice

**Fig 4:**
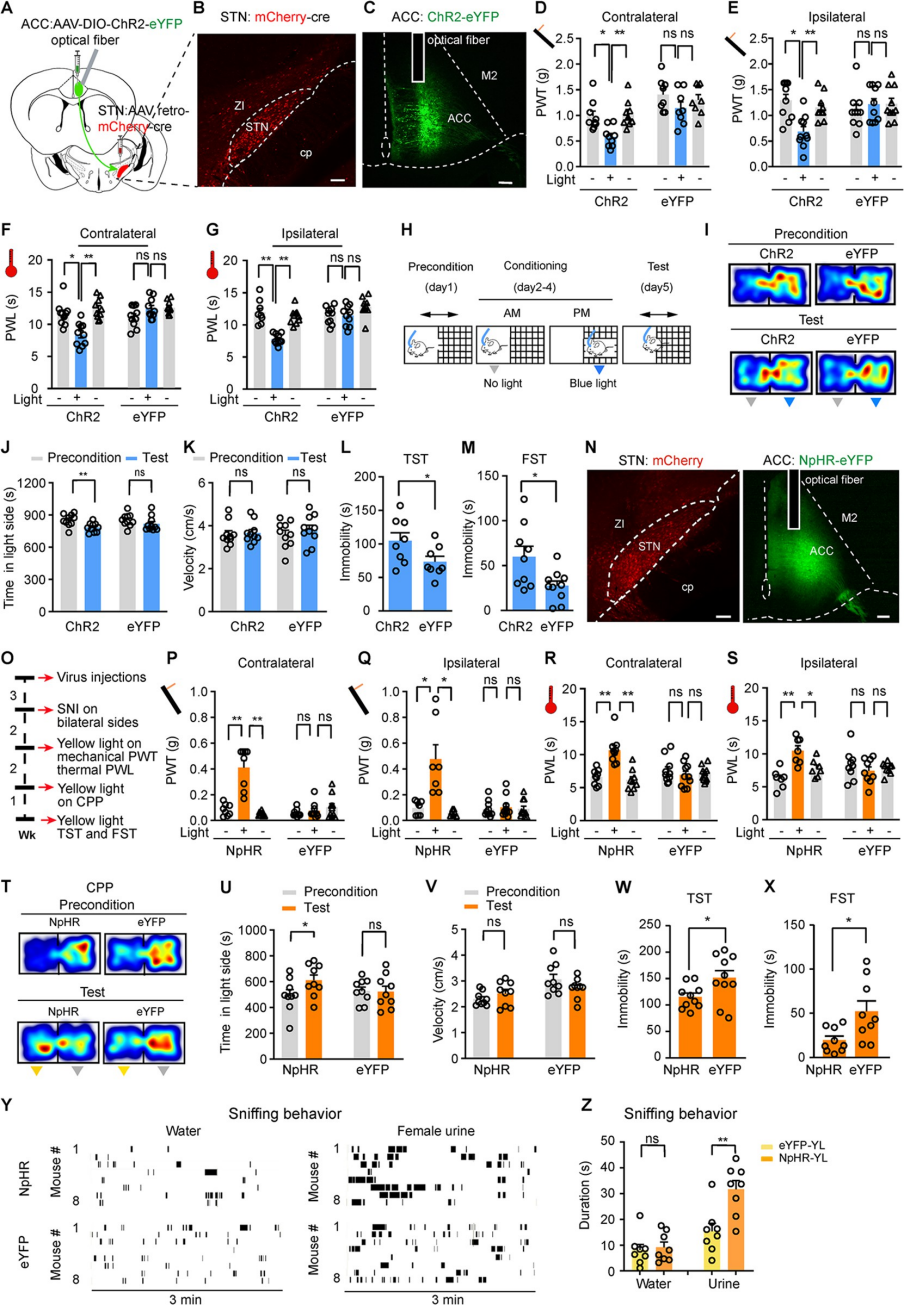
STN-projecting ACC neurons regulate pain thresholds, place preference, and depression-like behavior. **(A)** Schematic diagram of virus injections and optical fiber implantation for optogenetic activation of STN-projecting ACC neurons. (**B and C**) Representative images of retrograde transfection of STN-projecting ACC neurons with ChR2. **(D–G)** Effect of optogenetic activation of unilateral STN-projecting ACC neurons on mechanical PWT (**D:** F (_1, 17_) = 13.18, *P* = 0.0021; **E:** F (_1, 17_) = 8.38, *P* = 0.01; *n* = 9 ChR2 mice, *n* = 10 eYFP mice) and thermal PWL (**F**: F_(1, 18)_ = 5.08, *P* = 0.037; **G**: F_(1, 18)_ = 15.87, *P* = 0.0009; *n* = 10 mice in each group) on the contralateral and ipsilateral hind paws. **(H–K)** Schematic diagram (**H**), representative heat maps **(I)**, and quantification of time spent (**J**) and velocity (**K**) in the blue-light-paired chamber in the preconditioning and test sessions in ChR2 and eYFP mice. (**J)** Time, F_(1, 18)_ = 13.26, *P* = 0.0019. (**K)** Velocity, F_(1, 18)_ = 0.78, *P* = 0.39, *n* = 10 mice in each group. (**L and M)** Quantification of immobility time of ChR2 and eYFP mice during blue light illumination (473 nm, 20 Hz, 5 ms pulse width, 4 mW) of STN-projecting ACC neurons in the TST (**L,** *t* = 2.17, *P* = 0.03) and the FST (**M,** *t* = 2.59, *P* = 0.019). *n* = 9 mice in each group. **(N)** Example images of virus expression in the injection sites. (**O**) Time line of the experiments for panels (**N**, **P–X**). (**P–S**) Effect of optogenetic silencing of STN-projecting ACC neurons on mechanical PWT (**P**: F_(2, 32)_ = 19.33, *P* < 0.0001, *n* = 8 NpHR mice, *n* = 10 eYFP mice) (**Q**: F_(2, 32)_ = 17.43, *P* < 0.0001, *n* = 7 NpHR mice, *n* = 10 eYFP mice) and thermal PWL (**R**: F_(2, 34)_ = 11.85, *P* = 0.0001, *n* = 9 NpHR mice, *n* = 10 eYFP mice) (**S:** F_(2, 30)_ = 5.58, *P* = 0.0087, *n* = 8 NpHR mice, *n* = 9 eYFP mice). **(T–V)** Representative heat maps **(T)** and quantification of time spent (**U**) and velocity (**V**) in the yellow light (589 nm, constant 3 mW, 2-min episodes with 2-min intervals)-paired chamber in the preconditioning and test sessions for SNI mice (**U**: F_(1, 16)_ = 12.6, *P* = 0.0027; **V**: F_(1, 16)_ = 4.27, *P* = 0.055; *n* = 9 mice in each group). (**W** and **X**) Effect of optogenetic silencing of STN-projecting ACC neurons on immobility time in the TST (**W**, t = 2.28, *P* = 0.03, *n* = 10 mice) and FST (**X**, t = 2.65, *P* = 0.02, *n* = 9 mice). (**Y** and **Z**) Raster plots and summary showing water and female urine sniffing behavior in eYFP or NpHR mice subjected to SNI upon 3 min yellow light illumination of the ACC. (**Z**) F_(1, 28)_ = 29.82, *P* < 0.0001, *n* = 8 mice each group. *P < 0.05. **P < 0.01; two-way repeated measures ANOVA with Tukey’s post hoc analysis for **(D–G, J** and **K, P–S, U,** and **V)**; two-tailed paired *t* test for (**L, M, W**, and **X**). One-way ANOVA was used for **(Z)**. Scale bars: 100 μm. Source data can be found in the fifth worksheet of S1 Data. ACC, anterior cingulate cortex; FST, forced swim test; PWT, paw withdrawal threshold; SNI, spared nerve injury; STN, subthalamic nucleus; TST, tail suspension test.

ACC neurons are consistently suggested to participate in pain perception and neuropsychiatric disorders, including depression [34,37,38]. We tried to determine whether ACC-STN neurons, a group of ACC neurons, exert similar effect on pain-like and depression-like behavior. We transfected ChR2 into ACC-STN neurons by injecting AAV retro-hSyn-mCherry-Cre into the STN and injecting AAV-EF1α-DIO-ChR2-eYFP or AAV-EF1α-DIO-eYFP into the ipsilateral ACC (Figs 4A–4C and S6A–S6D). Immunofluorescence staining showed that most mCherry-labeled neurons in the ACC expressed a marker protein of glutamatergic neurons, vesicular glutamate transporter 1 (VgluT1) (S6E and S6F Fig). Using patch-clamp technique, we confirmed that blue light pulses evoked time-locked inward currents in ChR2-expressing ACC neurons, suggesting that this strategy allows for efficient manipulation of ACC-STN neurons (S6G and S6H Fig). In behavioral tests, we observed that optogenetic activation of ACC-STN neurons reduced mechanical and thermal thresholds on both hind paws in naïve mice (Fig 4D–4G). Note that mechanical and thermal thresholds in control eYFP mice were not affected by blue light illumination of the ACC (Fig 4D–4G). The data from ChR2 mice support that stimulation of ACC-STN neurons induces pain-like behaviors in naïve mice.

We employed conditioned place preference/aversion (CPP/CPA) paradigm to determine whether repetitive activation of ACC-STN neurons leads to CPP/CPA. On day 1, the mice were allowed for freely roaming in 2 chambers in CPP/CPA box for 20 min in the absence of light stimulation. In days 2 to 4, blue light pulses (473 nm, 20 Hz, 5 ms, 4 mW) (15 min/day) were delivered into the ACC when the mice were placed in the chamber with meshed floor (Fig 4H). On day 5, ChR2 mice displayed significant CPA, while control eYFP mice did not show either CPP or CPA (Fig 4I and 4J). We also observed that blue light illumination of the ACC did not alter the traveling velocity in the blue light-paired chamber in either ChR2 or eYFP mice (Fig 4K). These data exclude the possibility that CPA was confounded by altered locomotor activity.

To determine whether hyperactivity in ACC-STN neurons is sufficient to induce depression-like behaviors in naïve mice, we performed 2 routine assays including the tail suspension test (TST) (increased immobility) and forced swim test (FST) (increased immobility). We observed that optogenetic activation (473 nm, 5 ms pulses, 20 Hz, 4 mW) of ACC-STN neurons in mice significantly prolonged immobility time in TST and FST (Fig 4L and 4M). These data suggest that ACC-STN neurons are among ACC neurons triggering depression-like behaviors.

### Inhibition of ACC-STN neurons ameliorates hyperalgesia and depression-like behavior in neuropathic pain

**Fig 5:**
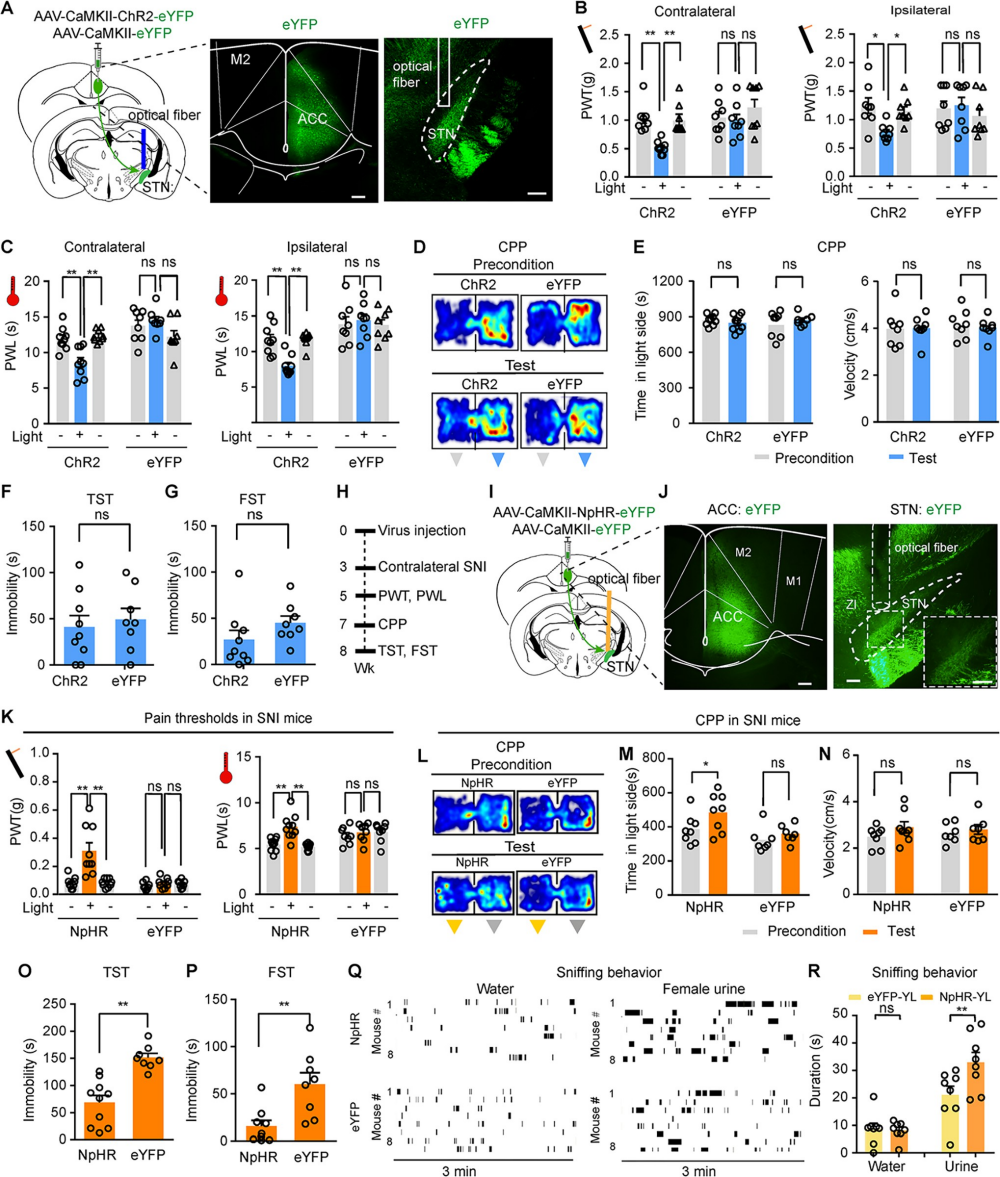
The ACC-STN projection modulates physiological pain thresholds and chronic pain. **(A)** Schematic diagram and representative images showing virus injection into the ACC and fiber implantation into the STN for optogenetic activation of the ACC-STN projection. **(B** and **C)** Effect of blue light illumination (473 nm, 20 Hz, 5 ms pulse width, 4 mW) of the ACC-STN projection on mechanical PWT and thermal PWL in naïve mice. (**B**) PWT. Contralateral: F_(2, 28)_ = 10.94, *P* = 0.0003; Ipsilateral: F_(2, 28)_ = 4.46, *P* = 0.02. (**C**) PWL. Contralateral: F_(2, 30)_ = 15.29, *P* < 0.0001; Ipsilateral: F_(2, 30)_ = 8.84, *P* = 0.001. *n* = 9 ChR2 mice, *n* = 8 eYFP mice. **(D** and **E)** Representative heat maps **(D)** and quantification of time spent (**E**, left) and velocity (**E**, right) in the blue light-paired chamber during the precondition and test sessions for ChR2 and eYFP mice. (**E**, left) Time, F_(1, 15)_ = 1.85, *P* = 0.19; (**E**, right) Velocity, F_(1, 15)_ = 0.003, *P* = 0.96; *n* = 9 ChR2 mice; *n* = 8 eYFP mice. (**F** and **G**) Quantification of immobility time of ChR2 and eYFP mice during blue light-illumination of the ACC-STN projection in the TST (**F**, t = 0.47, *P* = 0.65) and FST (**G**, t = 1.39, *P* = 0.18). *n* = 9 ChR2 mice, *n* = 8 eYFP mice. (**H**) Time line of experiments in (**I**–**P**). (**I**) Schematic diagram of virus injection and fiber implantation for optogenetic silencing of the ACC-STN projection. (**J**) Example images of NpHR-eYFP-labeled ACC neurons (left) and NpHR-eYFP-axonal fibers in the STN (right). Insertion in the right panel shows high magnification of NpHR-eYFP labeled axonal fibers in the STN. (**K**) Effect of yellow light (589 nm, constant, 3 mW)-illumination of the ACC-STN projection on mechanical PWT (F_(2, 30)_ = 10.24, *P* = 0.0004) and thermal PWL (F_(2, 30)_ = 15.5, *P* < 0.0001) in NpHR and eYFP mice 2 weeks after SNI surgery on the contralateral side. *n* = 9 NpHR mice, *n* = 8 eYFP mice. **(L–N)** Representative heat maps **(L)** and quantification of time spent (**M**) and velocity (**N**) in the yellow-light (589 nm, 3 mW, 2-min episodes with 2-min intervals)-paired chamber during the precondition and test sessions in NpHR and eYFP mice 4 weeks after SNI surgery. (**M)** Time, F_(1, 14)_ = 10.61, *P* = 0.0057; *n* = 8 mice in each group. (**N)** Velocity, F_(1, 14)_ = 2.15, *P* = 0.16; *n* = 8 mice in each group. (**O** and **P**) Quantification of immobility time of NpHR and eYFP mice 4 weeks after SNI surgery during yellow light-illumination of the ACC-STN projection in the TST (**O**, t = 5.1, *P* = 0.0001) and FST (**P**, t = 3.4, *P* = 0.004). *n* = 9 NpHR mice, *n* = 8 eYFP mice. (**Q**) Raster plots (left panels) and summary (right panel) of water and female urine sniffing behavior in eYFP or NpHR SNI mice upon 3 min yellow light illumination of the STN. F_(1, 28)_ = 47.34, *P* < 0.0001, *n* = 8 mice each group. * *P* < 0.05. ** *P* < 0.01; two-way ANOVA with Tukey’s post hoc analysis for **(B, C, E, K, M, N,** and **Q)**; two-tailed unpaired *t* test for **(F, G, O,** and **P)**. Scale bars: 100 μm. Source data can be found in the sixth worksheet of S1 Data. ACC, anterior cingulate cortex; FST, forced swim test; PWL, paw withdrawal latency; PWT, paw withdrawal threshold; SNI, spared nerve injury; STN, subthalamic nucleus; TST, tail suspension test.

The above data demonstrate that ACC-STN neurons are implicated in pain-like behaviors and negative emotions (Fig 4A–4M). These data prompted us to investigate whether inhibition of these neurons ameliorates hyperalgesia and depression-like behaviors in SNI mice. We used the same combinatorial optogenetic strategy shown in Fig 4A to selectively transfect NpHR into ACC-STN neurons and inhibit these neurons with yellow light (Figs 4N, S6G, and S6I). Although optogenetic inhibition (continuous pulse, 589 nm laser, 3 mW) of ACC-STN neurons did not affect mechanical and thermal thresholds in either hind paw in naïve mice (Figs 4O and S6J–S6M), photo-inhibition of ACC-STN neurons dramatically elevated mechanical and thermal thresholds on both hind paws in SNI mice (Figs 4P–5S). Since illumination of the ACC with yellow light in control eYFP mice did not change mechanical and thermal thresholds in SNI mice (Fig 4P–4S), our data indicate that ACC-STN neurons play an important role in the maintenance of hyperalgesia in SNI mice.

In a CPP paradigm described in Fig 4H, we found that photo-inhibition of ACC-STN neurons significantly increased the time spent in the light-paired chamber in SNI mice (Fig 4T–4V). Furthermore, in SNI mice with ACC-STN neurons expressing NpHR, yellow light illumination throughout the test session shortened immobility time in the TST and FST, compared with SNI mice with ACC-STN neurons expressing eYFP (Fig 4W and 4X). We further performed female urine sniffing test as a non-operant test for measuring anhedonia in depression-like behavior [39]. SNI mice with NpHR or eYFP transfected into ACC-STN neurons were subjected to sniffing test with water and urine of female mice. Time of female urine sniffing was significantly increased in NpHR mice upon yellow light illumination compared with that in eYFP mice, but time of water sniffing was similar between NpHR and eYFP mice (Fig 4Y and 4Z).

These results demonstrate that reversing hyperactivity in ACC-STN neurons is sufficient to mitigate hyperalgesia and depression-like behaviors in chronic pain.

### The modulation of pain thresholds and depression-like behaviors by ACC-STN projection in naïve and SNI mice

Besides the STN, ACC-STN neurons also projected to other nuclei (S7A–S7C Fig), and these projections may contribute to the behavioral results (Fig 4) following stimulation of ACC-STN neurons. Therefore, we subsequently investigated whether direct modulation of the ACC axonal terminals in the STN could exert effects similar to the modulation of ACC-STN neurons. To address this, we injected mice with AAV-CaMKII-ChR2-eYFP, AAV-CaMKII-NpHR-eYFP, or AAV-CaMKII-eYFP into the right ACC and implanted optical fiber in the right STN (Figs 5A, 5H–5J, and S8A–S8D). As expected, we observed that blue light stimulation (20 Hz, 5 ms pulse, 473 nm, 4 mW) of the ACC-STN projection reduced mechanical and thermal thresholds on both hind paws in ChR2 mice, but not in eYFP mice (Fig 5B and 5C). We performed the CPP paradigm described in Fig 4H and unexpectedly found that optogenetic stimulation of the ACC-STN projection did not induce preference or aversion to the light-paired chamber (Fig 5D and 5E). Similarly, optogenetic stimulation of the ACC-STN projection did not affect depression-like behaviors assessed with the TST and FST (Fig 5F and 5G). Therefore, the ACC-STN projection only replicates the effect of the activation of ACC-STN neurons on pain modulation but not that on aversion- and depression-like behaviors.

We then examined the behavioral outcomes following optogenetic inhibition of the ACC-STN projection in naïve and SNI mice. We observed that optogenetic inhibition of the ACC-STN projection (continuous light, 589 nm laser) did not alter mechanical and thermal thresholds on either hind paw in naïve mice (S8E–S8H Fig), but optogenetic inhibition of the ACC-STN projection replicated the effects of inhibition of ACC-STN neurons on several aspects in SNI mice, such as, a significant increase of mechanical paw withdrawal threshold (PWT) and thermal paw withdrawal latency (PWL) (Fig 5K), the establishment of CPP (Fig 5L–5N), and a dramatic attenuation of depression-like behaviors measured with the TST, FST, and female urine sniffing test (Fig 5O–5Q).

Together, these experiments support that the ACC-STN projection mediates the role of ACC-STN neurons in pain modulation in naïve mice, but in modulation of both pain-like and depression-like behaviors in SNI mice.

### The ACC-STN projection interplays with STN neurons to modulate pain thresholds and depression-like behavior

**Fig 6:**
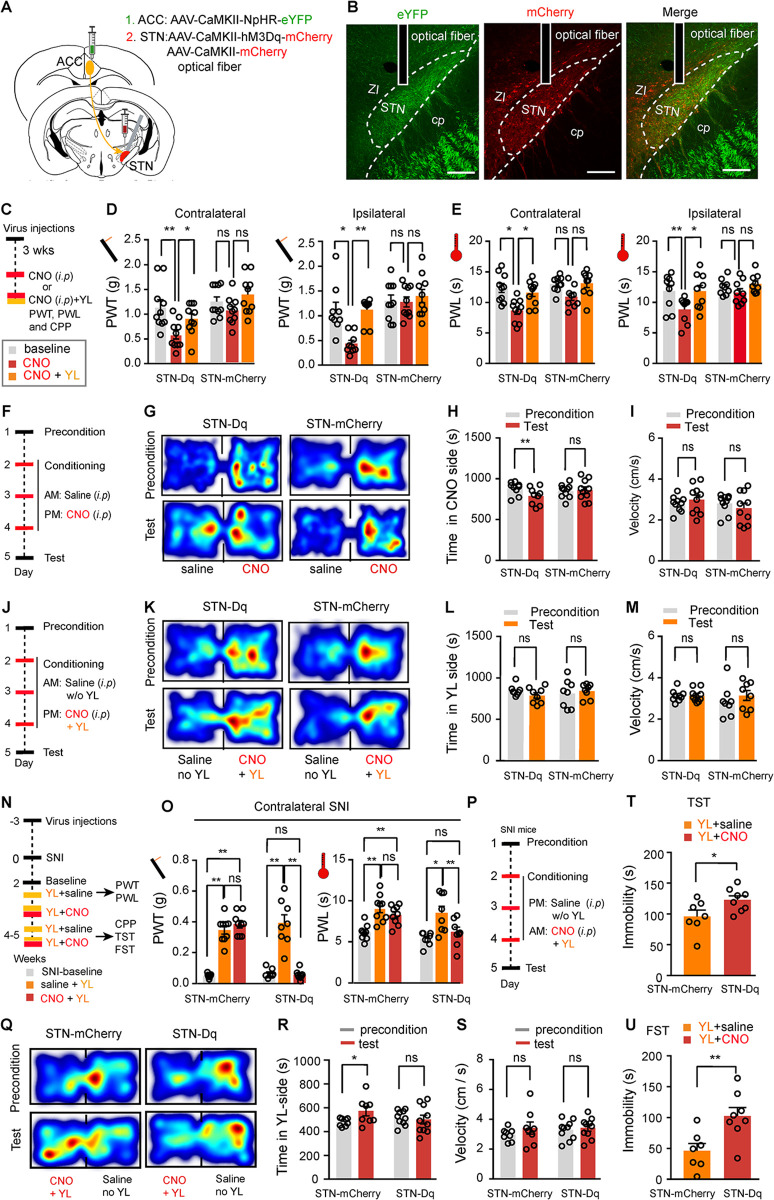
The ACC-STN projection regulates pain-like and depression-like behaviors by gating the activity of STN neurons. **(A)** Schematic diagram of virus injections for optogenetic silencing of the ACC-STN projection and chemogenetic activation of STN neurons. (**B**) Representative images of NpHR-eYFP-labeled axonal fibers (left) and hM3Dq-mCherry-labeled neuronal somata (middle) in the STN and the merged image (right). (**C**) Time line and experimental design for panels (**D** and **E**). (**D** and **E**) Effect of optogenetic silencing of the ACC-STN projection on CNO-activation of STN neurons-induced reduction of mechanical PWT and thermal PWL in naïve mice (**D**: Contralateral, F_(2, 36)_ = 3.20, *P* = 0.05; Ipsilateral, F_(2, 34)_ = 12.02, *P* = 0.0001) (**E**: Contralateral, F_(2, 34)_ = 12.82, *P* < 0.0001; Ipsilateral, F_(2, 34)_ = 13.24, *P* < 0.0001). *n* = 9 mice in STN(Dq)/ACC(NpHR) group, *n* = 10 mice in STN(mCherry)/ACC(NpHR) group. (**F**) Schematic diagram of CPP protocol for panels (**G–I**). (**G–I**) Representative heat maps **(G)** and quantification of time spent (**H**) and velocity (**I**) in CNO-paired chamber during the precondition and test sessions for STN-hM3Dq (Dq)- and STN-mCherry mice. (**H)** Time, F_(1, 17)_ = 5.38, *P* = 0.033. (**I**) Velocity, F_(1, 17)_ = 1.45, *P* = 0.25; *n* = 9 mice in STN(Dq) group, *n* = 10 mice in STN(mCherry) group. (**J**) Schematic diagram of CPP protocol for panels (**K–M**). (**K–M**) Representative heat maps **(K)** and quantification of time spent (**L**) and velocity (**M**) in CNO plus yellow light (YL)-paired chamber during the precondition and test sessions for STN(Dq)/ACC(NpHR)- and STN(mCherry)/ACC (NpHR) mice. (**L**) Time, F_(1, 14)_ = 2.09, *P* = 0.17. (**M**) Velocity, F_(1, 14)_ = 1.0, *P* = 0.33; *n* = 8 mice in each group. (**N**) Time line and experimental design for **(O–U)**. (**O**) Effect of optogenetic silencing of the ACC-STN projection with YL on CNO-activation of STN neurons-induced reduction in mechanical PWT and thermal PWL 2 weeks after the contralateral SNI surgery (PWT: treatment, F_(2, 30)_ = 84.6, *P* < 0.0001; PWL: treatment, F_(2, 30)_ = 24.26, *P* < 0.0001). *n* = 9 mice in ACC(NpHR)/STN(mCherry) group, *n* = 8 mice in ACC(NpHR)/STN(Dq) group. (**P**) Schematic diagram of CPP protocol for (**Q–S**). **(Q–S)** Representative heat maps **(Q)** and quantification of time spent (**R**) and velocity **(S)** in CNO plus YL-paired chamber during the precondition and test sessions in SNI mice. (**R)** Time, F_(1, 16)_ = 6.08, *P* = 0.025. (**S**) Velocity, F_(1, 16)_ = 0.51, *P* = 0.48; *n* = 8 mice in ACC(NpHR)/STN(mCherry) group, *n* = 10 mice in ACC(NpHR)/STN(Dq) group. (**T** and **U**) Quantification of immobility time of mice 4 weeks after SNI surgery during CNO activation of STN neurons and YL-illumination of the ACC-STN projection in the TST (**T**, *t* = 2.28, *P* = 0.04) and FST (**U**, *t* = 3.12, *P* = 0.0082). *n* = 7 mice in ACC(NpHR)/STN(mCherry) group, *n* = 8 mice in ACC(NpHR)/STN(Dq) group. * *P* < 0.05. ** *P* < 0.01; two-way repeated measures ANOVA with Tukey’s post hoc analysis for **(D**, **E**, **H**, **I**, **L**, **M**, **O**, **R**, **and S)**; two-tailed unpaired *t* test for (**T** and **U**). Scale bars: 100 μm. Source data can be found in the seventh worksheet of S1 Data. ACC, anterior cingulate cortex; CNO, clozapine-N-oxide; CPP, conditioned place preference; FST, forced swim test; PWL, paw withdrawal latency; PWT, paw withdrawal threshold; SNI, spared nerve injury; STN, subthalamic nucleus.

Our previous studies demonstrate that stimulation of STN neurons reduces pain threshold in naïve mice [19,20]. We then tested whether inhibition of the ACC-STN projection is enough to affect the activity of STN neurons and mitigates hyperalgesia induced by stimulation of STN neurons. To address this question, we injected AAV-CaMKII-hM3Dq-mCherry or AAV-CaMKII-mCherry into the STN and AAV-CaMKII-NpHR-eYFP into the ACC, and implanted optical fiber into the STN (Figs 6A, 6B, S9A, and S9B). Thus, we were able to activate STN neurons by intraperitoneal administration of CNO and inhibit the ACC-STN projection by yellow light illumination of the STN. Our patch-clamp recording from STN neurons expressing hM3Dq-mCherry in brain slices showed that bath perfusion of CNO (3 μM) increased firing rate in these neurons (S9C and S9D Fig).

We measured mechanical and thermal thresholds before and after intraperitoneal injection of CNO with or without yellow light illumination of the STN (Fig 6C). We found that CNO injection significantly decreased mechanical PWT and thermal PWL on both hind paws in hM3Dq mice, but not in mCherry mice (Fig 6D and 6E); subsequent optogenetic inhibition of the ACC-STN projection elevated both mechanical PWT and thermal PWL in hM3Dq mice received CNO injection; optogenetic inhibition of the ACC-STN projection had no effect on either mechanical or thermal thresholds in mCherry mice (Fig 6D and 6E). Therefore, inhibition of the ACC-STN projection is sufficient to attenuate hyperalgesia following hyperactivity of STN neurons.

Considering the difference in temporal resolution between optogenetic inhibition/stimulation and CNO-based chemogenetic modulation, we made some modifications in the CPA paradigm. hM3Dq or mCherry mice freely roamed in the CPA chamber for 20 min on day 1. On day 2 through day 4, these mice were intraperitoneally injected with saline and placed to 1 chamber for 20 min, and 4 h later, the mice were intraperitoneally injected with CNO and placed to the other chamber for 20 min. On day 5, the mice were allowed roaming freely in 2 chambers, and the time in each chamber was recorded (Fig 6F). We found that CNO injection induced significant place aversion for CNO-paired side in hM3Dq mice but not in mCherry mice (Fig 6G–6I). In another set of experiment, during 3 days’ conditioning, we applied yellow light illumination of the STN when mice stayed in CNO injection-paired chamber (Fig 6J). We observed that CNO-induced CPA in hM3Dq mice was blocked by optogenetic inhibition of the ACC-STN projection (Fig 6K and 6L). Optogenetic inhibition of the ACC-STN projection did not alter the time spent in the CNO-paired chamber in mCherry mice (Fig 6K and 6L). We found that neither CNO injection nor CNO injection with yellow light illumination of the STN changed the traveling velocity in hM3Dq or mCherry mice (Fig 6I and 6M). Therefore, inhibition of the ACC-STN projection is an effective approach to mitigate aversion induced by hyperactivity of STN neurons.

We next explored whether analgesic effect of inhibition of the ACC-STN projection in SNI mice is related to regulation of STN neurons. First, we compared c-Fos-positive STN neurons in SNI mice with and without receiving optogenetic inhibition of the ACC-STN projection. We found that optogenetic inhibition of the ACC-STN projection significantly reduced the number of c-Fos-positive STN neurons in SNI mice (S10A–S10C Fig). Second, we examined the effect of optogenetic inhibition of the ACC-STN projection on pain thresholds and CPP in SNI mice with and without chemogenetic activation of STN neurons. We observed that activation of STN neurons with CNO prevented the analgesic effects of inhibition of the ACC-STN projection in SNI mice (Fig 6O). In contrast, analgesic effect of optogenetic inhibition of the ACC-STN projection was not affected by CNO injection in mCherry mice in which STN neurons were not activated by CNO (Fig 6O). Activation of STN neurons also eliminated CPP and antidepressant effects of inhibition of the ACC-STN projection in SNI mice (Fig 6Q–6U). Therefore, the analgesic and antidepressant effect of the inhibition of the ACC-STN projection in SNI mice is relevant to its regulation of STN neurons.

## Discussion

The comorbidity of chronic pain and psychiatric disorders, such as depression, has long been recognized clinically [40,41], and preclinical studies have reported depression-like behaviors in animal models of chronic pain [6–8,11]. These findings suggest that common neural circuit bases may exist for these pathological states. In this study, we focused on the ACC-STN pathway. Using viral vector-assisted circuit tracing, in vivo Ca^2+^ imaging, optogenetic and chemogenetic modulation, and electrophysiological and morphological assay in mice, we demonstrated that inhibition of the ACC-STN pathway had benefit to not only hyperalgesia but also depression-like behaviors in chronic pain mice. First, inhibition of ACC neurons attenuated responses of STN neurons to mechanical, thermal, and aversive stimulation. Second, optogenetic inhibition of ACC-STN neurons and the ACC-STN projection alleviated pain sensitivity, established CPP, and mitigated depression-like behaviors in SNI mice. Third, the analgesic and antidepressant effect of the inhibition of the ACC-STN projection in SNI mice was blocked by chemogenetic activation of STN neurons. These data suggest that the ACC-STN pathway plays an important role in the maintenance of hyperalgesia and negative emotion in chronic pain. Additionally, our findings support a notion that the hyperactivity of STN neurons is important for the manifestation of hyperalgesia and depression-like behavior in chronic pain; the ACC-STN pathway is one but not the only synaptic inputs contributing to the hyperactivity of STN neurons in chronic pain; only when normal activity of STN neurons is restored, are symptoms of chronic pain effectively mitigated.

The basal ganglia, including the STN, relays nociceptive signals to brain nuclei implicated in pain processing and modulation [19,20,42]. Our recent studies demonstrated that the STN and its projections are involved in both somatosensory and emotional aspects of chronic pain [7,19,20]. The ACC has similar function as the STN in these aspects [25–27]. Using optogenetic and patch-clamp techniques, we confirmed that ACC glutamatergic neurons monosynaptically innervated STN glutamatergic neurons. Although the ACC is a source of hyperdirect pathway to the STN, which plays an important role in motor control [31,43,44], it is unclear whether the ACC-STN pathway is involved in the modulation of pain threshold and emotional processing by the STN. We found that enhancing the activity of STN neurons by optogenetic stimulation of the ACC-STN projection led to hyperalgesia, but did not cause CPA and depression-like behavior in naïve mice; chemogenetic inhibition of ACC glutamatergic neurons suppressed activation of STN neurons induced by pain-like and aversion-like stimulation. Therefore, the hyperdirect pathway provides a bridge between the ACC and STN conferring regulatory effects on pain thresholds. These results expand the roles of the hyperdirect pathway.

Photostimulation of ACC glutamatergic neurons establishes CPA and induces depression-like behavior in naïve animals [5,34,35,37]. Similar to these studies, we observed that optogenetic activation of ACC-STN neurons was sufficient to elicit aversion- and depression-like behavior in naïve mice. However, activation of the ACC-STN projection did not elicit aversion- and depression-like behaviors in naive mice. In contrast, chemogenetic activation of STN neurons results in aversion- and depression-like behavior in naïve mice. These inconsistencies may suggest the complexity of the ACC-STN pathway. Together with previous studies [5,10], this study shows that besides the STN, ACC-STN neurons project to the basolateral amygdala, bed nucleus of stria terminalis, nucleus accumbens, caudate putamen, claustrum, periaqueductal gray, thalamic nuclei, and superior colliculus, etc., it suggests that these projections may work together to regulate emotion. This may explain why the ACC-STN projection did not regulate emotions like ACC-STN neurons. The activation of ACC axonal terminals in the STN mobilizes a proportion of STN neurons which may be less than optogenetic stimulation of the STN does. This may explain why the effects of STN neuron stimulation differ from stimulation of the ACC-STN projection. These results suggest that in the ACC-STN pathway, the information in ACC-STN neurons may not completely transmit to the STN, and STN neurons are influenced by other brain regions in addition to the ACC.

Hyperactivity in STN neurons has been reported in several pain states [7,19,20]. Although the STN receives projections from many brain regions implicated in various pain states [29,45], the contribution of these brain regions to the hyperactivity in STN neurons has not been profoundly studied. Our recent study reported that reduced synaptic inputs from GABAergic neurons in the substantia nigra pars reticulata (SNr) may contribute to the hyperactivity of STN neurons in mice with chronic pain [7]. The present study shows that the excitability of ACC-STN neurons and synaptic strength of the ACC-STN projection in chronic pain mice were enhanced. We observed that optogenetic activation of either ACC-STN neurons or the ACC-STN projection induced hypersensitivity to mechanical and thermal stimuli and CPA in naïve mice. These data suggest that enhanced ACC-STN pathway may be sufficient to cause hyperalgesia and depression-like behavior in chronic pain. In contrast, we found that inhibition of this projection mitigated hyperalgesia and depression-like behavior in chronic pain, suggesting that the enhancement of the ACC-STN pathway may be necessary for these phenotypes in chronic pain. These evidence underscores a significant role of the ACC-STN pathway in facilitating and maintaining hyperalgesia and depression-like behavior in chronic pain.

We have demonstrated that the activity of STN neurons is elevated in mice with comorbid chronic pain and depression [7] and in mice exposed to acute stress [17]. Parolari and colleagues [46] reported that repeated inhibition of a subpopulation of STN neurons expressing Gabrr3 gene suppressed grooming in a mouse model for obsessive–compulsive disorder (OCD). These findings collectively suggest that increased activity of STN neurons may be a common neuronal mechanism underlying emotional dysfunctions, such as depression, occurred in chronic pain, acute stress, and OCD.

While our study provides evidence showing the involvement of the ACC-STN pathway in chronic pain and depression, it is important to acknowledge that the experiments have some limitations. First, as sex differences have been observed in relation to chronic pain and emotional disorders [9,47,48], future studies should investigate whether sex acts as a biological variable in the effects of the ACC-STN pathway on hyperalgesia and depression-like behaviors in chronic pain. Second, in fiber photometry recording, we examined the responses of ACC and STN neurons to pain, aversive, and anxiety-like stimulation, while in neuromodulation experiments, we focused on pain threshold and depression-like behaviors. Anxiety and depression, 2 major psychiatric disorders with a comorbidity of about 50% [48], affect each other [48], but their neural circuit mechanisms may not be identical [49–51]. Further investigations are needed to address the role of the ACC-STN pathway in representation and modulation of anxiety and depression.

In summary, our study demonstrates that the ACC-STN pathway plays a significant role in regulating pain and emotion with somewhat difference among its components, including ACC-STN neurons, the ACC-STN projection, and STN neurons. Notably, a significant interplay may exist between the ACC-STN projection and STN neurons to control the manifestations in comorbid chronic pain and depression. This study supports that the ACC-STN pathway may be a therapeutic target to treat comorbid chronic pain and depression.

## Materials and methods

### Ethics statement

The care and use of animals and the experimental protocols (No. 202207S123) used in this study were approved by the Institutional Animal Care and Use Committee and the Office of Laboratory Animal Resources of Xuzhou Medical University under the Regulations for the Administration of Affairs Concerning Experimental Animals (1988) in China.

### Animals

Wild-type C57BL/6J (WT) mice were purchased from the animal facility at Xuzhou Medical University. The mice were group-housed (no more than 5 per cage) in a temperature- and humidity-controlled housing facility on a 12-h light/dark cycle with ad libitum access to food and water. Male mice at least 8 weeks old were used in the experiments. Efforts were made to minimize animal suffering and to reduce the number of animals used.

### Viral vectors

AAV-CaMKII-ChR2-eYFP, AAV-CaMKII-eYFP, AAV-CaMKII-GCaMP6s, AAV-CaMKII-NpHR3.0-eYFP, AAV retro-hSyn-Cre-eGFP, AAV retro-hSyn-Cre-mCherry, and AAV-EF1α-DIO-eYFP were purchased from Brain VTA (Wuhan, China). AAV-CaMKII-hM4Di-mCherry, AAV-CaMKII-hM3Dq-mCherry, AAV-EF1α-DIO-ChR2-eYFP, AAV-EF1α-DIO-eYFP, and AAV-EF1α-DIO-NpHR3.0-eYFP were purchased from Brain Case (Wuhan, China). The viral titers are 2–5 × 10^12^ (viral genomes per ml) for AAV2/9.

### Stereotaxic surgeries and viral injection

Mice were deeply anesthetized with isoflurane (3% for induction, 1.5% for maintenance) (RWD Life Science Co., Shenzhen, China), placed on a heating pad, and stabilized on a stereotaxic apparatus (RWD Life Science Co., Shenzhen, China). Small holes were drilled in the skull above brain regions of interest. Injections (120 nl of virus per site at 50 nl/min) were made using an automatic microinjection pump (KD Scientific, Holliston, Massachusetts, United States of America).

The viral vectors were injected into the following coordinates (relative to the Bregma): STN (AP, −1.85 mm; ML, 1.48 mm; DV, 4.75 mm) and ACC (AP, +0.85 mm; ML, 0.4 mm; DV, 1.85 mm). Optical fiber implants (200 μm in diameter, NA 0.37) (Inper, Hangzhou, China) were placed 200 μm above (for optogenetic manipulation) or in (for fiber photometry recording) the injection site and were fixed to the skull with dental cement. Mice with virus injections and optical fiber implants were allowed to recover for at least 3 weeks before electrophysiological recordings and morphological assays. Viral expression and the position of fiber implants in each mouse were confirmed histologically after the termination of the experiments. We only included mice with viral expression confined to the STN or ACC and optical fibers in the right places for either optogenetic modulation or fiber photometry recording.

Meloxicam (4 mg/kg) (Aladdin Biochemical Technology, Shanghai, China) was administered subcutaneously once per day for 3 days for postoperative pain relief.

### Fiber photometry

A fiber photometry instrument (ThinkerTech, Nanjing, China) [6,18,20,52] was used to monitor GCaMP6 and eYFP signals in ACC and STN neurons. We adjusted the instrument by setting the excitation light to 50 μW and the gain to a level that gave a background signal of 3 units. The input optical cable was then connected to the optical implant in the mouse brain. To evaluate responses of STN and ACC neurons to mechanical, thermal, and emotional stimulation, we designated 3 s GCaMP6 and eYFP signal prior to the stimulation as the baseline value (F_0_) and calculated the mean and standard deviation (SD) of F_0_. We used these parameters to transform GCaMP6 and eYFP signal (F) into the z-score ((F–Mean of F_0_)/SD of F_0_) and measured the area under the curve (AUC, 12 s post stimulation) of the z-score plot to quantify the response of ACC neurons and STN neurons to sensory and emotional stimulation.

### Optogenetic manipulation

For ChR2-mediated optogenetic stimulation, 473-nm laser pulses (5 ms, 20 Hz, 4 mW) were delivered. For NpHR-mediated optogenetic inhibition, a 3 mW 589-nm laser was kept on continuously for 1 to 2 min (constant, 3 mW). All optogenetic manipulations were performed unilaterally in the right hemisphere. Therefore, the contralateral and ipsilateral sides refer to the left and the right sides, respectively.

### von Frey filament test

Individual mice were acclimatized for at least 1 h in a test compartment on a wide gauge wire mesh supported by an elevated platform. The von Frey filaments with fiber force between 0.01 and 2 g were used to measure mechanical PWT of both hind paws. The 50% threshold was determined with the up-down method [7,53].

### Thermal nociception threshold

Individual mice were acclimatized for at least 1 h in a test compartment on a glass surface. Thermal PWLs in both hind paws were measured with a plantar anesthesia tester (Boerni, Tianjin, China) [7,54].

### Spared nerve injury (SNI)

Chronic neuropathic pain model was established with spared nerve injury of the sciatic nerve according to a previously reported protocol [7,55]. Mice were deeply anesthetized using isoflurane (3% for induction, 1.5% for maintenance) (RWD Life Science Co., Shenzhen, China). The fur in the surgical area, extending from the knee to the hip, was shaved, and the skin was sterilized with 75% alcohol. A longitudinal incision was made in the shaved region, allowing for blunt dissection of the biceps femoris muscle to expose the sciatic nerve and its branches (sural, common peroneal, and tibial nerves). Two nylon sutures 3 mm apart were tightly ligated around the common peroneal and tibial nerves, and the nerves between the sutures were subsequently cut and removed. The mice were then allowed to recover on a heating pad.

Mice that did not receive nerve ligation and severing were used as sham controls. Pain thresholds were assessed using von Frey filaments and a heating beam targeting the skin area innervated by the sural nerve.

### Conditioned place preference (CPP) test

The CPP was performed in a custom-made two-chamber box (length × width × height: 40 × 20 × 30 cm^3^): the right chamber had vertical black-and-white stripes on the walls and a smooth floor, whereas the left chamber had horizontal black-and-white stripes on the walls and a mesh floor. The CPP test was performed according to a previous study with some modifications [20].

Day 1 was the preconditioning test (precondition) day. Mice were given free access to the 2 chambers and the time that the mice spent in each chamber was recorded. On days 2 to 4, the mice were restricted to 1 chamber for 20 min and received optogenetic modulation. At least 4 h later, the mice were restricted to the other chamber for 20 min without optogenetic modulation. On day 5 (around 18 h after the last conditioning), the mice were allowed to freely explore the 2 chambers for 20 min and the time spent in each chamber was recorded. On the precondition and test days, the animal’s movement was video-tracked and analyzed online or offline with an EthoVision XT video tracking software (Noldus Information Technology, Wageningen, the Netherlands) [56,57]. We calculated the time spent in the light-paired side on the precondition and test days. Mice were not used if they spent more than 75% of the total time in 1 chamber on the pre-test day.

### Measurement of depression-like behaviors

TST, FST, and female urine sniffing test have been used for assessing depression-like behaviors in rodents [6,7,39]. A longer immobility time in the TST and FST refers to despair, while a shorter time spent sniffing female urine refers to anhedonia in male mice.

### Tail suspension test (TST)

A mouse was suspended by taping its tail onto a horizontal bar 50 cm above the floor. The mouse was allowed to hang undisturbed for 6 min and its behavior was video-recorded. The total duration that the mouse remained immobile in the last 5 min was used to evaluate depression-like behavior.

### Forced swim test (FST)

Mice were placed in a 1 liter glass beaker filled with 26°C water. The movement of the mice was recorded for 5 min using a camera placed beside the beaker, and the immobility time was measured.

### Female urine sniffing test

This is a sex-related reward-seeking behavior [39]. Male mice were singly housed for 1 week before testing. In the test day, male mice in their home cage were first exposed for 3 min to a cotton tip dipped in tap water; 45 min later, male mice were exposed for 3 min to another cotton tip dipped in fresh urine collected from female mice in the estrus phase. The total times of water and female urine sniffing were measured.

### Brain slice electrophysiology

Brain slice electrophysiological recording was conducted with minor modifications according to previously reported methods [6,20,57]. Coronal slices (300 μm thick) containing the ACC and parasagittal slices (300 μm thick) containing the STN were prepared using a vibratome (Leica VT-1200S, Nussloch, Germany) in an ice-cold modified sucrose-based artificial cerebral spinal fluid (sACSF) saturated with 95% O_2_/5% CO_2_ (carbogen), containing (in mM) 85 NaCl, 75 sucrose, 2.5 KCl, 1.25 NaH_2_PO_4_, 4.0 MgCl_2_, 0.5 CaCl_2_, 24 NaHCO_3_, and 25 glucose. The brain slices were transferred into carbogenated sACSF at 32°C and allowed to recover for 60 min, and then placed in normal carbogenated ACSF containing (mM) 125 NaCl, 2.5 KCl, 1.2 NaH_2_PO_4_, 1.2 MgCl_2_, 2.4 CaCl_2_, 26 NaHCO_3_, and 11 glucose at 26°C for at least 30 min prior to use.

Neurons in brain slices were visualized under an upright microscope (FN-1, Nikon, Tokyo, Japan), equipped with a CCD-camera (Flash 4.0 LTE, Hamamatsu, Hamamatsu City, Japan). Whole-cell patch-clamp recordings were obtained using a patch-clamp setup composed of a dual-channel MultiClamp 700B amplifier, a Digidata 1550B analog-to-digital converter, and pClamp 10.7 software (Molecular Devices, San Jose, California, USA). The patch electrodes had a resistance of 4 to 6 MΩ when filled with a low-chloride intrapipette solution containing (in mM) 135 K gluconate, 0.2 EGTA, 0.5 CaCl_2_, 10 HEPES, 2 Mg-ATP, and 0.1 GTP, pH: 7.2; osmolarity: 290 to 300 mOsm. Neurons with a holding current larger than −50 pA and a resting membrane potential more depolarized than −40 mV were excluded from the analysis. All recordings were performed at 32 ± 1°C.

Light-evoked excitatory postsynaptic currents (eEPSCs) in the presence of tetrodotoxin (TTX, 1 μM) and 4-aminopyridine (4-AP, 0.3 mM) were recorded at −50 mV. To confirm whether glutamatergic connections were involved, CNQX (20 μM) were bath-applied. Firing in response to current injections (1 s, 20 to 200 pA steps with a 20 pA increment and a 30 s inter-sweep interval) were recorded in the current-clamp mode.

For light-evoked responses, blue light (460 nm, 2 mW) or yellow light (560 nm, 2 mW) was delivered through an optical fiber (200 μm, NA 0.37) connected to a PlexBright LED light source (Plexon, Hong Kong, China).

### Histology

Mice were sacrificed in a CO_2_ chamber and then subjected to cardiac perfusion with phosphate-buffered saline (PBS), followed by 4% paraformaldehyde (PFA) in PBS. Mouse brains were removed and post-fixed in 4% PFA overnight at 4°C. Brain samples were place into 30% sucrose in PBS until sank for cryoprotection, then imbedded in OCT, froze to −17°C, and cut into 30 μm sections with a Leica CM1950 cryostat (Nussloch, Germany). The brain sections were mounted onto glass slides. For immunostaining, brain sections were incubated in a blocking buffer containing 5% donkey serum and 0.1% Triton X-100 for 90 min at room temperature. Then, the sections were incubated with primary antibody diluted in blocking buffer for 24 h at 4°C (rabbit anti-c-Fos IgG, 1:2,000, Cell Signaling Technology; Rabbit anti-Vglut1 IgG, 1:750, Proteintech Group). After washing 3 times (10 min each) in PBS, the sections were incubated with secondary antibodies (Alexa 488- or Alexa 555-conjugated donkey anti-rabbit IgG, Jackson ImmunoResearch) for 90 min at room temperature. The sections were washed 3 times (10 min each) in PBS, dried in the dark, and then cover-slipped in mounting medium (Meilunbio, Dalian, China).

The sections were imaged with a confocal microscope (LSM 880, Zeiss) and the images were processed with ImageJ (NIH) [58].

### Chemicals

4-aminopyridine (4-AP), 6-Cyano-7-nitro-quinoxaline-2, 3-dione disodium salt hydrate (CNQX), and tetrodotoxin (TTX) were purchased from Tocris.

### Statistical analysis

GraphPad Prism 7.0 was used for statistical analyses. Clampfit 10.7 (Molecular Devices) was used for analysis of electrophysiological and GCaMP6 data. Figures were prepared with Adobe Illustrator CS6. All summarized data are expressed as mean ± SEM. Two-tailed paired or unpaired *t* tests were used for comparison of a parameter between 2 groups if data were normally distributed. One-way or two-way, ordinary or repeated measures ANOVAs followed by Tukey’s post hoc analysis were used for multiple comparisons. If the equal-variance assumptions were not valid, statistical significance was evaluated with the Mann–Whitney test or ANOVA rank tests. The mean and SEM, *n* (the number of animals), statistical test, and *t*, F, and *P* values are reported in the figure legends. A value of *P* < 0.05 was considered statistically significant. The minimal number of mice used in each experiment was calculated in a priori power analysis (StatMate 2.0) and the power of each experiment was set to 0.8. The sample sizes in each experiments are larger than the minimal numbers.

## Supporting information

S1 Fig Neurons in the anterior cingulate cortex are hyperactive in SNI mice. **Related to Fig 1.** (**A**) Schematic diagram of SNI surgery (upper panel) and time line of experiments for measurement of pain-like and depression-like behaviors. **(B** and **C)** Time course of mechanical **(B)** and thermal **(C)** thresholds after SNI surgery. (B) F_(1, 12)_ = 212.9, *P* < 0.0001. **(C)** F_(1, 12)_ = 475.9, *P* < 0.0001. *n* = 6 sham mice, *n* = 8 SNI mice. PWT: paw withdrawal threshold; PWL: paw withdrawal latency. **(D** and **E)** Immobility time in the TST **(D**, *t* = 2.54, *P* = 0.026, *n* = 6 in sham, *n* = 8 in SNI) and the FST (**E**, *t* = 3.38, *P* = 0.0055, *n* = 6 sham mice, *n* = 8 SNI mice**)**. **(F)** Comparison of time spending sniffing water and female urine between sham and SNI mice. SNI mice showed reduced time spent sniffing female urine compared with sham mice (F_(1, 28)_ = 24.15, *P* < 0.0001, *n* = 8 mice in each group). **(G** and **H)** Representative images **(G)** and quantification **(H)** of c-Fos-(+) neurons in the ACC 4 weeks after sham operation or SNI surgery on the right side (*t* = 8.9, *P* < 0.0001; *n* = 5 mice in each group). ** *P* < 0.01; two-way repeated measures ANOVA with Tukey’s post hoc analysis for **(B** and **C);** two-tailed *t* test for **(D**, **E**, and **H);** one-way ANOVA was used for **(F)**. * *P* < 0.05; ** *P* < 0.01; ns, not significant. Scale bars: 200 μm. Source data can be found in the first worksheet of S2 Data.
(TIFF)

S2 Fig eYFP signal in ACC neurons does not change with pain-like and emotion-like behavior. **Related to Fig 2. (A)** Schematic diagram showing virus (AAV-CaMKII-eYFP) injection and fiber implantation into the ACC for fiberphotometry. **(B–D)** Heat maps (**B**), average traces (**C**), and quantification (**D,** t = 0.67, *P* = 0.51) of eYFP signal in the ACC of naïve mice in response to von Frey filament (vF) and thermal stimulation of hind paws. (**E–G**) Heat maps (**E**), average traces (**F**), and quantification (**G,** t = 0.31, *P* = 0.67) of eYFP signal in the ACC of naïve or SNI mice expressing eYFP in the ACC in response to 1 s air puff on the face. (**H–J**) Heat maps (**H**), average traces (**I**), and quantification (**J,** F_(2, 57)_ = 2.69, *P* = 0.076) of eYFP signal in the ACC of naïve or SNI mice expressing eYFP in the ACC during exploration of the open or closed arms in the elevated plus maze. Two-tailed *t* test for panels (**D**, **G**, *n* = 5 mice.); one-way ANOVA with Tukey’s post hoc analysis for (**J**, *n* = 5 mice in control and SNI groups). ns, not significant. Source data can be found in the second worksheet of S2 Data.
(TIFF)

S3 Fig Subthalamic neurons are activated by nociceptive and aversive stimulation. **Related to Fig 3. (A)** Representative images showing virus injection (AAV-CaMKII-mCherry into the ACC and AAV-CaMKII-GCaMP6s into the STN) and fiber implantation into the STN. **(B–M)** Heat maps (**B, E, H, and K**), averaged traces (**C**, **F**, **I**, and **L**), and quantification **(D**, **G**, **J**, **and M)** of GCaMP6s signal in the STN of mice receiving von Frey (2 g vF) or 50°C thermal stimulation of hind paws after intraperitoneal injection of either saline or CNO (3 mg/kg). **(D)** *t* = 0.073, *P* = 0.94. **(G)** *t* = 0.033, *P* = 0.97. (**J**) *t* = 0.49, *P* = 0.63. **(M)** *t* = 0.30, *P* = 0.77. (**N–P**) Heat maps (**N**), average traces (**O**), and quantification (**P**, t = 0.50, *P* = 0.62) of GCaMP6s signal in the STN of mice exposed to 1 s air puff to the face after intraperitoneal injection of saline or CNO. CNO or the same volume of saline was applied 45 min prior to GCaMP6s signal recording. Dashed lines in panels indicate application of stimuli. Two-tailed *t* test for (**D**, **G**, **J**, **M**, and **P**, *n* = 5 mice.). ns: not significant. Source data can be found in the third worksheet of S2 Data.
(TIFF)

S4 Fig Chemogenetic inhibition of ACC neurons attenuates the responses of STN neurons to mechanical, thermal, and aversive stimulation in SNI mice. **Related to Fig 3. (A–X)** Heat maps (**A, D, G, J, M, P, S, and V**), average traces (**B, E, H, K, N, Q, T, and W**), and quantification (**C, F, I, L, O, R, U, and X**) of GCaMP6s signal in the STN of mice with mCherry or hM4Di expression in the ACC in SNI mice. All mice received von Frey (0.16 g) or thermal (48°C) stimulation of hind paws after intraperitoneal injection of saline or CNO (3 mg/kg). **(C)** *t* = 0.21, *P* = 0.84. **(F)** *t* = 2.69, *P* = 0.01. (**I**) *t* = 0.33, *P* = 0.74. (**L**) *t* = 2.82, *P* = 0.0072. **(O)** *t* = 1.24, *P* = 0.22. **(R)** *t* = 3.16, *P* = 0.00027. (**U**) *t* = 0.45, *P* = 0.64. (**X**) *t* = 2.77, *P* = 0.008. **(Y–Z2)** Heat maps (**Y**), average traces (**Z1**), and quantification (**Z2**, *t* = 2.15, *P* = 0.03, unpaired two-tailed *t* test) of GCaMP6s signal in the STN of SNI mice with hM4Di expression in the ACC in response to 1 s air puff onto the faces after intraperitoneal injection of saline or CNO. CNO or the same volume of saline was applied 45 min prior to fiber photometry recording of GCaMP6s signal. Dashed lines in panels indicate application of stimuli. Two-tailed *t* test for (**C**, **F**, **I**, **L, O, R, U, X**, and **Z2**, *n* = 5 mice). * *P* < 0.05, ** *P* < 0.01; ns, not significant. Source data can be found in the fourth worksheet of S2 Data.
(TIFF)

S5 Fig Activity of subthalamic neurons in naïve and SNI mice changes differently during exploration in the elevated plus maze. **Related to Fig 3. (A–L)** Heat maps (**A, D, G, and J**), average traces (**B, E, H, and K**), and quantification (**C, F, I, and L**) of GCaMP6s signal in the STN of naïve mice with mCherry or hM4Di expression in the ACC. All mice explored in the EPM 45 min after intraperitoneal injection of saline or CNO (3 mg/kg). **(C)** *t* = 0.081, *P* = 0.94. **(F)** *t* = 0.08, *P* = 0.57. (**I**) *t* = 0.19, *P* = 0.85. (**L**) *t* = 0.42, *P* = 0.68. **(M–O)** The hM4Di mice received SNI surgery and fiber photometry recording from the STN were performed 2 weeks later. Changes of GCaMP6s signal in the STN are presented as heat maps **(M)**, average traces **(N)**, and AUC of GCaMP6s signal **(O)** when the mice entered into the open arms and closed arms 45 min after intraperitoneal administration of saline or CNO. **(O)** Open arms: t = 0.54, *P* = 0.59, saline vs. CNO; closed arms: t = 2.37, *P* = 0.022, Saline vs. CNO_._ Dashed lines in panels indicate application of stimuli. Two-tailed *t* test for panels (**C**, **F**, **I**, and **L** *n* = 5 mice). One-way ANOVA for **(O)**. ns, not significant. Source data can be found in the fifth worksheet of S2 Data.
(TIFF)

S6 Fig Verification of virus expression and function in STN-projecting ACC neurons. **Related to Fig 4. (A** and **B)** Representative image (left) and schematic diagram (right) showing AAV retro-hSyn-mCherry-Cre injection into the STN **(A)**, AAV-EF1α-DIO-ChR2-eYFP injection and optical fiber implantations into the ACC **(B)** for optogenetic manipulation. **(C** and **D)** Example images of mCherry expression in the STN and eYFP expression in mCherry(+) STN projecting ACC neurons. (**E** and **F**) Representative images (**E**) and quantification (**F**) of mCherry+ neurons (red) labeled with VGluT1-antibody (magenta) (*n* = 4 mice). (**G**) Schematic diagram showing recording of ChR2- or NpHR-labeled STN projecting ACC neurons. (**H**) Representative trace (left) and quantification (right) of corresponding inward currents recorded from ChR2-eYFP-labeled ACC neurons in response to 20 Hz blue light stimulation. *n* = 7 cells from 3 mice. (**I**) Representative trace (left) and quantification (right) of corresponding outward currents recorded from NpHR-eYFP-labeled ACC neurons in response to 1 s yellow light stimulation. *n* = 7 cells from 3 mice. **(J–M)** Effect of optogenetic silencing of STN-projecting ACC neurons on mechanical PWT (**J:** Contralateral, F_(1, 16)_ = 0.022, *P* = 0.88; **K**: Ipsilateral, F_(1, 16)_ = 0.12, *P* = 0.73; *n* = 9 mice) and thermal PWL (**L**: Contralateral, F_(1, 16)_ = 0.025, *P* = 0.88, *n* = 9 mice; **M**: Ipsilateral, F_(1, 16)_ = 1.09, *P* = 0.31, *n* = 9 mice) on both hind paws. Open circles in the right panel of **(A)** and **(B)** indicate the locations of virus injection and optical fiber implantations, respectively. *P < 0.05. Two-way repeated measures ANOVA with Tukey’s post hoc analysis for **(J–M)**. Scale bars: 100 μm. Source data can be found in the sixth worksheet of S2 Data.
(TIFF)

S7 Fig Neuronal tracing of the projections of ACC-STN neurons. **Related to Fig 5. (A)** Schematic diagram showing locations of virus injection. AAV retro-hSyn-mCherry-Cre was injected into the STN and AAV-EF1α-DIO-eYFP was injected into the ACC to label ACC-STN neurons and their projecitons. (**B** and **C**) Representative images and quantification for distribution of eYFP-labeled axonal fibers in brain regions. *n* = 4 mice. Acb: nucleus accumbens; BLA: basolateral amygdala; BST: bed nucleus of the stria terminalis; Cl: claustram; CM: centromedial thalamus; cp: cerebral peduncle; CPu: caudate putamen; InWh: Intermediate white layer of the superior colliculus; LPAG: lateral periaqueductal gray; LS: lateral septum; PV: paraventricular thalamic nucleus; MD: mediodorsal thalamus; IMA: intramedullary thalamus; LD: laterodorsal thalamus; ml: medial lemniscus; Pn: Pontine nucleus; PnO: pontine reticular oral part; Po: posterior thalamus; VL: ventrolateral thalamus; VM: ventromedial thalamus; VPM and VPL: ventroposterior thalamus medial and lateral part; Re: reuniens thalamus; SNr: substantial nigra pars reticulata; STN: subthalamic nucleus; SuG, InG and DpG: superficial, intermediate and deep gray layer of the superior colliculus; Zi: zona incerta. Scale bars: 100 μm. Source data can be found in the seventh worksheet of S2 Data.
(TIFF)

S8 Fig The behavioral effects of optogenetic inhibition of the ACC-STN projection. **Related to Fig 6. (A–C)** Schematic diagram of virus injection **(A** and **B)** and optical fiber implantation **(C)** for optogenetic activation of the ACC-STN projection. Right panels in **(B)** and **(C)** summarize locations of virus injections into the ACC and optical fiber implantations into the STN, respectively. (**D**) A representative image (left) and locations of optical fiber implantations (right) in the STN for optogenetic inhibition of the ACC-STN projection. (**E–H**) Effect of yellow light illumination (589 nm, continuous, 3 mW) of the ACC-STN projection on mechanical PWT and thermal PWL in naïve mice. (**E**) F_(2, 28)_ = 2.35, *P* = 0.11; **(F)** F_(2, 28)_ = 0.67, *P* = 0.52; *n* = 8 mice in each group; (**G**) F_(2, 30)_ = 0.12, *P* = 0.89; (**H**) F_(2, 30)_ = 1.75, *P* = 0.19. *n* = 9 NpHR mice, *n* = 8 eYFP mice. Two-way repeated measures ANOVA with Tukey’s post hoc analysis for **(E–H)**. ns, not significant. Scale bars: 100 μm. Source data can be found in the eighth worksheet of S2 Data.
(TIFF)

S9 Fig Verification of expression and function of chemogenetic virus in the STN. **Related to Fig 6. (A)** A representative image and summary for the locations of optical fiber implants in the STN for optogenetic inhibition of the ACC-STN projections and virus injection sites for chemogenetic activation of STN neurons. (**B**) Left: Schematic diagram of virus injections for chemogenetic activation of STN neurons; right: representative image of hM3Dq-mCherry-labeled STN neurons. (**C** and **D**) Representative traces and quantification of evoked action potentials before, during (5 min), and 10 min after termination of CNO (3 μM) perfusion. Firing rate: F_(1.2, 6.001)_ = 17.17, *P* = 0.0051. *n* = 6 neurons. One-way repeated measures ANOVA with Tukey’s post hoc analysis for **(D)**. * *P* < 0.05; *P* < 0.01. Scale bars: 100 μm. Source data can be found in the ninth worksheet of S2 Data.
(TIFF)

S10 Fig c-Fos expression in the STN of SNI mice is reduced by optogenetic inhibition of the ACC-STN projection. **Related to Fig 6. (A)** Schematic diagram of virus injection and fiber implantation for optogenetic silencing of the ACC-STN projection. Yellow light was delivered into the STN for 20 min (589 nm, continuous, 3 mW, 2 min episodes with 30 s intervals) in NpHR- and eYFP-expressing mice 4 weeks after SNI surgery. The mice were sacrificed 1 h after yellow light illumination for c-Fos-staining. (**B and C**) Representative images (**B**) and quantification (**C**) of c-Fos(+) STN neurons. **(C)** F_(2, 32)_ = 12.54, *P* < 0.0001. One-way ANOVA with Tukey’s post hoc analysis. ** *P* < 0.01, *n* = 12 slices from 4 mice in each group. Scale bars: 100 μm. Source data can be found in the tenth worksheet of S2 Data.
(TIFF)

S1 Table Changes of GCaMP6s signal in the STN in response to pain stimulation. Related to Figs 3 and S4. Source data can be found in S3 Data.
(DOCX)

S2 Table Changes of GCaMP6s signal in the STN in response to air puff and during exploration in the elevated plus maze. Related to Figs 3, S4, and S5. Source data can be found in S3 Data.
(DOCX)

S1 Data Source data for Figs 1–6. (XLSX)

S2 Data Source data for S1–S10 Figs. (XLSX)

S3 Data Source data for S1 and S2 Tables. (XLSX)

## Abbreviations

ACC: anterior cingulate cortex
ACSF: artificial cerebral spinal fluid
AUC: area under the curve
CNO: clozapine-N-oxide
CPA: conditioned place aversion
CPP: conditioned place preference
EPM: elevated plus maze
EPSC: excitatory postsynaptic current
FST: forced swim test
OCD: obsessive–compulsive disorder
PD: Parkinson’s disease
PWL: paw withdrawal latency
PWT: paw withdrawal threshold
SD: standard deviation
SNI: spared nerve injury
STN: subthalamic nucleus
TST: tail suspension test
WT: wild-type

## References

[1] Bair MJ, Robinson RL, Katon W. Kroenke K Depression and pain comorbidity: a literature review. Arch Intern Med. 2003;163(20):2433–2445. doi: 10.1001/archinte.163.20.2433 14609780

[2] Rayner L, Hotopf M, Petkova H, Matcham F, Simpson A, McCracken LM. Depression in patients with chronic pain attending a specialised pain treatment centre: prevalence and impact on health care costs. Pain. 2016;157(7):1472–1479. doi: 10.1097/j.pain.0000000000000542 26963849 PMC4912238

[3] Arnow BA, Hunkeler EM, Blasey CM, Lee J, Constantino MJ, Fireman B, et al. Comorbid depression, chronic pain, and disability in primary care. Psychosom Med. 2006;68(2):262–268. doi: 10.1097/01.psy.0000204851.15499.fc 16554392

[4] Meerwijk EL, Ford JM. Weiss SJ Brain regions associated with psychological pain: implications for a neural network and its relationship to physical pain. Brain Imaging Behav. 2013;7(1):1–14. doi: 10.1007/s11682-012-9179-y 22660945

[5] Becker LJ, Fillinger C, Waegaert R, Journee SH, Hener P, Ayazgok B. et al. The basolateral amygdala-anterior cingulate pathway contributes to depression-like behaviors and comorbidity with chronic pain behaviors in male mice. Nat Commun. 2023;14(1):2198. doi: 10.1038/s41467-023-37878-y 37069164 PMC10110607

[6] Ji YW, Shen ZL, Zhang X, Zhang K, Jia T, Xu X, et al. Plasticity in ventral pallidal cholinergic neuron-derived circuits contributes to comorbid chronic pain-like and depression-like behaviour in male mice. Nat Commun. 2023;14(1):2182. doi: 10.1038/s41467-023-37968-x 37069246 PMC10110548

[7] Yin C, Jia T, Luan Y, Zhang X, Xiao C, Zhou C. A nigra-subthalamic circuit is involved in acute and chronic pain states. Pain. 2022;163(10):1952–1966. doi: 10.1097/j.pain.0000000000002588 35082251

[8] Llorca-Torralba M, Camarena-Delgado C, Suarez-Pereira I, Bravo L, Mariscal P, Garcia-Partida JA, et al. Pain and depression comorbidity causes asymmetric plasticity in the locus coeruleus neurons. Brain. 2022;145(1):154–167. doi: 10.1093/brain/awab239 34373893 PMC8967092

[9] Shen Z, Li W, Chang W, Yue N, Yu J. Sex differences in chronic pain-induced mental disorders: Mechanisms of cerebral circuitry. Front Mol Neurosci. 2023;16(2):1102808. doi: 10.3389/fnmol.2023.1102808 36891517 PMC9986270

[10] Vogt BA. Pain and emotion interactions in subregions of the cingulate gyrus. Nat Rev Neurosci. 2005;6(7):533–544. doi: 10.1038/nrn1704 15995724 PMC2659949

[11] Zhou W, Jin Y, Meng Q, Zhu X, Bai T, Tian Y, et al. A neural circuit for comorbid depressive symptoms in chronic pain. Nat Neurosci. 2019;22(10):1649–1658. doi: 10.1038/s41593-019-0468-2 31451801

[12] Pellaprat J, Ory-Magne F, Canivet C, Simonetta-Moreau M, Lotterie JA, Radji F, et al. Deep brain stimulation of the subthalamic nucleus improves pain in Parkinson’s disease. Parkinsonism Relat Disord. 2014;20(6):662–664. doi: 10.1016/j.parkreldis.2014.03.011 24685343

[13] Mostofi A, Morgante F, Edwards MJ, Brown P, Pereira EAC. Pain in Parkinson’s disease and the role of the subthalamic nucleus. Brain. 2021;144(5):1342–1350. doi: 10.1093/brain/awab001 34037696 PMC7612468

[14] Sun Y, Wang Z, Hu K, Mo Y, Cao P, Hou X, et al. alpha and theta oscillations in the subthalamic nucleus are potential biomarkers for Parkinson’s disease with depressive symptoms. Parkinsonism Relat Disord. 2021;90(9):98–104. doi: 10.1016/j.parkreldis.2021.07.023 34419805

[15] Chaudhuri KR, Healy DG, Schapira AH. National Institute for Clinical E Non-motor symptoms of Parkinson’s disease: diagnosis and management. Lancet Neurol. 2006;5(3):235–245. doi: 10.1016/S1474-4422(06)70373-8 16488379

[16] Parker T, Huang Y, Gong C, Chen Y, Wang S, Green AL, et al. Pain-Induced Beta Activity in the Subthalamic Nucleus of Parkinson’s Disease. Stereotact Funct Neurosurg. 2020;98(3):193–199. doi: 10.1159/000507032 32348997

[17] Jia T, Chen J, Wang YD, Xiao C, Zhou CY. A subthalamo-parabrachial glutamatergic pathway is involved in stress-induced self-grooming in mice. Acta Pharmacol Sin. 2023. doi: 10.1038/s41401-023-01114-6 37322164 PMC10618182

[18] Wu H, Yan X, Tang D, Gu W, Luan Y, Cai H, et al. Internal States Influence the Representation and Modulation of Food Intake by Subthalamic Neurons. Neurosci Bull. 2020;36(11):1355–1368. doi: 10.1007/s12264-020-00533-3 32567027 PMC7674539

[19] Luan Y, Tang D, Wu H, Gu W, Wu Y, Cao JL, et al. Reversal of hyperactive subthalamic circuits differentially mitigates pain hypersensitivity phenotypes in parkinsonian mice. Proc Natl Acad Sci U S A. 2020;117(18):10045–10054. doi: 10.1073/pnas.1916263117 32312820 PMC7211985

[20] Jia T, Wang YD, Chen J, Zhang X, Cao JL, Xiao C, et al. A nigro-subthalamo-parabrachial pathway modulates pain-like behaviors. Nat Commun. 2022;13(1):7756. doi: 10.1038/s41467-022-35474-0 36522327 PMC9755217

[21] Kolling N, Wittmann MK, Behrens TE, Boorman ED, Mars RB, Rushworth MFS, et al. Value, search, persistence and model updating in anterior cingulate cortex. Nat Neurosci. 2016;19(10):1280–1285. doi: 10.1038/nn.4382 27669988 PMC7116891

[22] de Lima MAX, Baldo MVC, Oliveira FA. Canteras NS The anterior cingulate cortex and its role in controlling contextual fear memory to predatory threats. Elife. 2022;11(1):e67007. doi: 10.7554/eLife.67007 34984975 PMC8730726

[23] Jhang J, Lee H, Kang MS, Lee HS, Park H, Han J-H. Anterior cingulate cortex and its input to the basolateral amygdala control innate fear response. Nat Commun. 2018;9(1):2744. doi: 10.1038/s41467-018-05090-y 30013065 PMC6048069

[24] Xiao X, Ding M, Zhang YQ. Role of the Anterior Cingulate Cortex in Translational Pain Research. Neurosci Bull. 2021;37(3):405–422. doi: 10.1007/s12264-020-00615-2 33566301 PMC7954910

[25] Bliss TV, Collingridge GL, Kaang BK, Zhuo M. Synaptic plasticity in the anterior cingulate cortex in acute and chronic pain. Nat Rev Neurosci. 2016;17(8):485–496. doi: 10.1038/nrn.2016.68 27307118

[26] Drevets WC, Savitz J, Trimble M. The subgenual anterior cingulate cortex in mood disorders. CNS Spectr. 2008;13(8):663–681. doi: 10.1017/s1092852900013754 18704022 PMC2729429

[27] Apkarian AV, Bushnell MC, Treede RD, Zubieta JK. Human brain mechanisms of pain perception and regulation in health and disease. Eur J Pain. 2005;9(4):463–484. doi: 10.1016/j.ejpain.2004.11.001 15979027

[28] Benarroch EE. Subthalamic nucleus and its connections: Anatomic substrate for the network effects of deep brain stimulation. Neurology. 2008;70(21):1991–1995. doi: 10.1212/01.wnl.0000313022.39329.65 18490619

[29] Emmi A, Antonini A, Macchi V, Porzionato A, De Caro R. Anatomy and Connectivity of the Subthalamic Nucleus in Humans and Non-human Primates. Front Neuroanat. 2020;14(4):13. doi: 10.3389/fnana.2020.00013 32390807 PMC7189217

[30] Hamani C, Florence G, Heinsen H, Plantinga BR, Temel Y, Uludag K, et al. Subthalamic Nucleus Deep Brain Stimulation: Basic Concepts and Novel Perspectives. eNeuro. 2017;4 (5):ENEURO.0140-0117.2017. doi: 10.1523/ENEURO.0140-17.2017 28966978 PMC5617209

[31] Haynes WI, Haber SN. The organization of prefrontal-subthalamic inputs in primates provides an anatomical substrate for both functional specificity and integration: implications for Basal Ganglia models and deep brain stimulation. J Neurosci. 2013;33(11):4804–4814. doi: 10.1523/JNEUROSCI.4674-12.2013 23486951 PMC3755746

[32] Kummer KK, Mitric M, Kalpachidou T, Kress M. The Medial Prefrontal Cortex as a Central Hub for Mental Comorbidities Associated with Chronic Pain. Int J Mol Sci. 2020;21(10):3440. doi: 10.3390/ijms21103440 32414089 PMC7279227

[33] Monosov IE, Haber SN, Leuthardt EC, Jezzini A. Anterior Cingulate Cortex and the Control of Dynamic Behavior in Primates. Curr Biol. 2020;30(23):R1442–R1454. doi: 10.1016/j.cub.2020.10.009 33290716 PMC8197026

[34] Barthas F, Sellmeijer J, Hugel S, Waltisperger E, Barrot M, Yalcin I. The anterior cingulate cortex is a critical hub for pain-induced depression. Biol Psychiatry. 2015;77(3):236–245. doi: 10.1016/j.biopsych.2014.08.004 25433903

[35] Sellmeijer J, Mathis V, Hugel S, Li XH, Song Q, Chen Q-Y, et al. Hyperactivity of Anterior Cingulate Cortex Areas 24a/24b Drives Chronic Pain-Induced Anxiodepressive-like Consequences. J Neurosci. 2018;38(12):3102–3115. doi: 10.1523/JNEUROSCI.3195-17.2018 29463643 PMC6596067

[36] Greicius MD, Flores BH, Menon V, Glover GH, Solvason HB, Kenna H, et al. Resting-state functional connectivity in major depression: abnormally increased contributions from subgenual cingulate cortex and thalamus. Biol Psychiatry. 2007;62(5):429–437. doi: 10.1016/j.biopsych.2006.09.020 17210143 PMC2001244

[37] Barthas F, Humo M, Gilsbach R, Waltisperger E, Karatas M, Leman S, et al. Cingulate Overexpression of Mitogen-Activated Protein Kinase Phosphatase-1 as a Key Factor for Depression. Biol Psychiatry. 2017;82(5):370–379. doi: 10.1016/j.biopsych.2017.01.019 28359564

[38] Koga K, Descalzi G, Chen T, Ko HG, Lu J, Li S, et al. Coexistence of Two Forms of LTP in ACC Provides a Synaptic Mechanism for the Interactions between Anxiety and Chronic Pain. Neuron. 2015;86(4):1109. doi: 10.1016/j.neuron.2015.05.016 28898636

[39] Fang X, Jiang S, Wang J, Bai Y, Kim CS, Blake D, et al. Chronic unpredictable stress induces depression-related behaviors by suppressing AgRP neuron activity. Mol Psychiatry. 2021;26(6):2299–2315. doi: 10.1038/s41380-020-01004-x 33432188 PMC8272726

[40] Woo AK. Depression and Anxiety in Pain. Rev Pain. 2010;4(1):8–12. doi: 10.1177/204946371000400103 26527193 PMC4590059

[41] Mullins PM, Yong RJ, Bhattacharyya N. Associations between chronic pain, anxiety, and depression among adults in the United States. Pain Pract. 2023;23(6):589–594. doi: 10.1111/papr.13220 36881021

[42] Borsook D, Upadhyay J, Chudler EH, Becerra L. A key role of the basal ganglia in pain and analgesia—insights gained through human functional imaging. Mol Pain. 2010;6(5):27. doi: 10.1186/1744-8069-6-27 20465845 PMC2883978

[43] Bogacz R, Larsen T. Integration of reinforcement learning and optimal decision-making theories of the basal ganglia. Neural Comput. 2011;23(4):817–851. doi: 10.1162/NECO_a_00103 21222528

[44] Cavanagh JF, Wiecki TV, Cohen MX, Figueroa CM, Samanta J, Sherman SJ, et al. Subthalamic nucleus stimulation reverses mediofrontal influence over decision threshold. Nat Neurosci. 2011;14(11):1462–1467. doi: 10.1038/nn.2925 21946325 PMC3394226

[45] Jeon H, Lee H, Kwon DH, Kim J, Tanaka-Yamamoto K, Yook SJ, et al. Topographic connectivity and cellular profiling reveal detailed input pathways and functionally distinct cell types in the subthalamic nucleus. Cell Rep. 2022;38(9):110439. doi: 10.1016/j.celrep.2022.110439 35235786

[46] Parolari L, Schneeberger M, Heintz N, Friedman JM. Functional analysis of distinct populations of subthalamic nucleus neurons on Parkinson’s disease and OCD-like behaviors in mice. Mol Psychiatry. 2021;26(11):7029–7046. doi: 10.1038/s41380-021-01162-6 34099874

[47] Munce SE, Stewart DE. Gender differences in depression and chronic pain conditions in a national epidemiologic survey. Psychosomatics. 2007;48(5):394–399. doi: 10.1176/appi.psy.48.5.394 17878497

[48] Kalin NH. The Critical Relationship Between Anxiety and Depression. Am J Psychiatry. 2020;177(5):365–367. doi: 10.1176/appi.ajp.2020.20030305 32354270

[49] Gold AL, Abend R, Britton JC, Behrens B, Farber M, Ronkin E, et al. Age Differences in the Neural Correlates of Anxiety Disorders: An fMRI Study of Response to Learned Threat. Am J Psychiatry. 2020;177(5):454–463. doi: 10.1176/appi.ajp.2019.19060650 32252541 PMC9078083

[50] Gray JP, Muller VI, Eickhoff SB, Fox PT. Multimodal Abnormalities of Brain Structure and Function in Major Depressive Disorder: A Meta-Analysis of Neuroimaging Studies. Am J Psychiatry. 2020;177(5):422–434. doi: 10.1176/appi.ajp.2019.19050560 32098488 PMC7294300

[51] Spellman T, Liston C. Toward Circuit Mechanisms of Pathophysiology in Depression. Am J Psychiatry. 2020;177(5):381–390. doi: 10.1176/appi.ajp.2020.20030280 32354265 PMC7643194

[52] Wang D, Liu P, Mao X, Zhou Z, Cao T, Xu J, et al. Task-Demand-Dependent Neural Representation of Odor Information in the Olfactory Bulb and Posterior Piriform Cortex. J Neurosci. 2019;39(50):10002–10018. doi: 10.1523/JNEUROSCI.1234-19.2019 31672791 PMC6978954

[53] Zhou C, Luo ZD. Nerve injury-induced calcium channel alpha-2-delta-1 protein dysregulation leads to increased pre-synaptic excitatory input into deep dorsal horn neurons and neuropathic allodynia. Eur J Pain. 2015;19(9):1267–1276. doi: 10.1002/ejp.656 25691360 PMC4539283

[54] Tang DL, Luan YW, Zhou CY, Xiao C. D2 receptor activation relieves pain hypersensitivity by inhibiting superficial dorsal horn neurons in parkinsonian mice. Acta Pharmacol Sin. 2021;42(2):189–198. doi: 10.1038/s41401-020-0433-3 32694753 PMC8027812

[55] Decosterd I, Woolf CJ. Spared nerve injury: an animal model of persistent peripheral neuropathic pain. Pain. 2000;87(2):149–158. doi: 10.1016/S0304-3959(00)00276-1 10924808

[56] Fan JP, Zhang X, Han Y, Ji Y, Gu WX, Wu HC, et al. Subthalamic neurons interact with nigral dopaminergic neurons to regulate movement in mice. Acta Physiol (Oxf). 2023;237(3):e13917. doi: 10.1111/apha.13917 36598331

[57] Ji YW, Zhang X, Fan JP, Gu WX, Shen ZL, Wu HC, et al. Differential remodeling of subthalamic projections to basal ganglia output nuclei and locomotor deficits in 6-OHDA-induced hemiparkinsonian mice. Cell Rep. 2023;42(3):112178. doi: 10.1016/j.celrep.2023.112178 36857188

[58] Schneider CA, Rasband WS, Eliceiri KW. NIH Image to ImageJ: 25 years of image analysis. Nat Methods. 2012;9(7):671–675. doi: 10.1038/nmeth.2089 22930834 PMC5554542

