# Supplementary figures and images for: The anterior cingulate cortex controls the hyperactivity in subthalamic neurons in male mice with comorbid chronic pain and depression
Source: PLoS Biol. 2024 Feb 22;22(2):e3002518. doi: 10.1371/journal.pbio.3002518 (PMC10883538; doi:10.1371/journal.pbio.3002518)

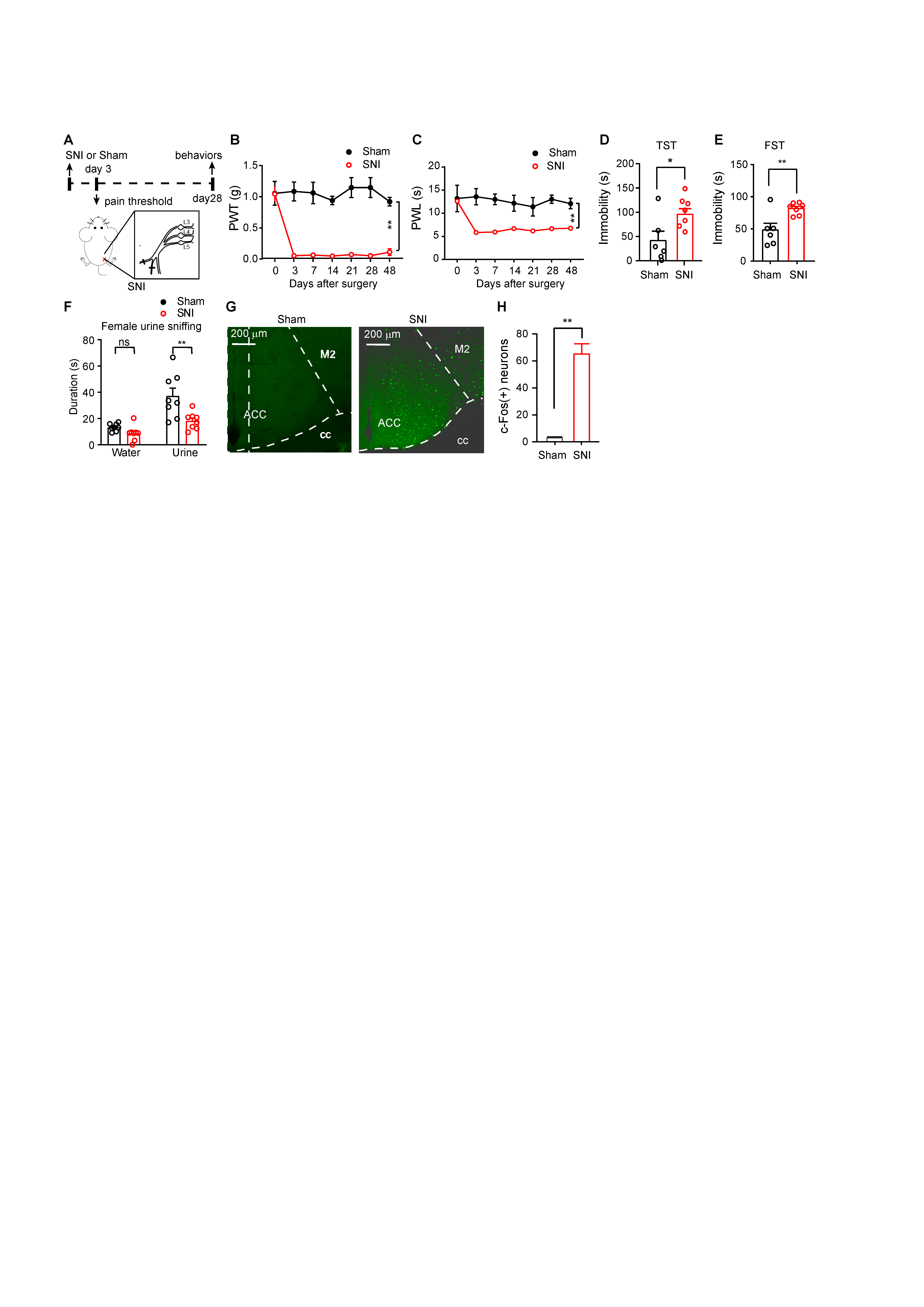

Supplement: S1 Fig — Related to Fig 1. (A) Schematic diagram of SNI surgery (upper panel) and time line of experiments for measurement of pain-like and depression-like behaviors. (B and C) Time course of mechanical (B) and thermal (C) thresholds after SNI surgery. (B) F(1, 12) = 212.9, P < 0.0001. (C) F(1, 12) = 475.9, P < 0.0001. n = 6 sham mice, n = 8 SNI mice. PWT: paw withdrawal threshold; PWL: paw withdrawal latency. (D and E) Immobility time in the TST (D, t = 2.54, P = 0.026, n = 6 in sham, n = 8 in SNI) and the FST (E, t = 3.38, P = 0.0055, n = 6 sham mice, n = 8 SNI mice). (F) Comparison of time spending sniffing water and female urine between sham and SNI mice. SNI mice showed reduced time spent sniffing female urine compared with sham mice (F(1, 28) = 24.15, P < 0.0001, n = 8 mice in each group). (G and H) Representative images (G) and quantification (H) of c-Fos-(+) neurons in the ACC 4 weeks after sham operation or SNI surgery on the right side (t = 8.9, P < 0.0001; n = 5 mice in each group). ** P < 0.01; two-way repeated measures ANOVA with Tukey’s post hoc analysis for (B and C); two-tailed t test for (D, E, and H); one-way ANOVA was used for (F). * P < 0.05; ** P < 0.01; ns, not significant. Scale bars: 200 μm. Source data can be found in the first worksheet of S2 Data. (TIFF) [file pbio.3002518.s001.tiff]

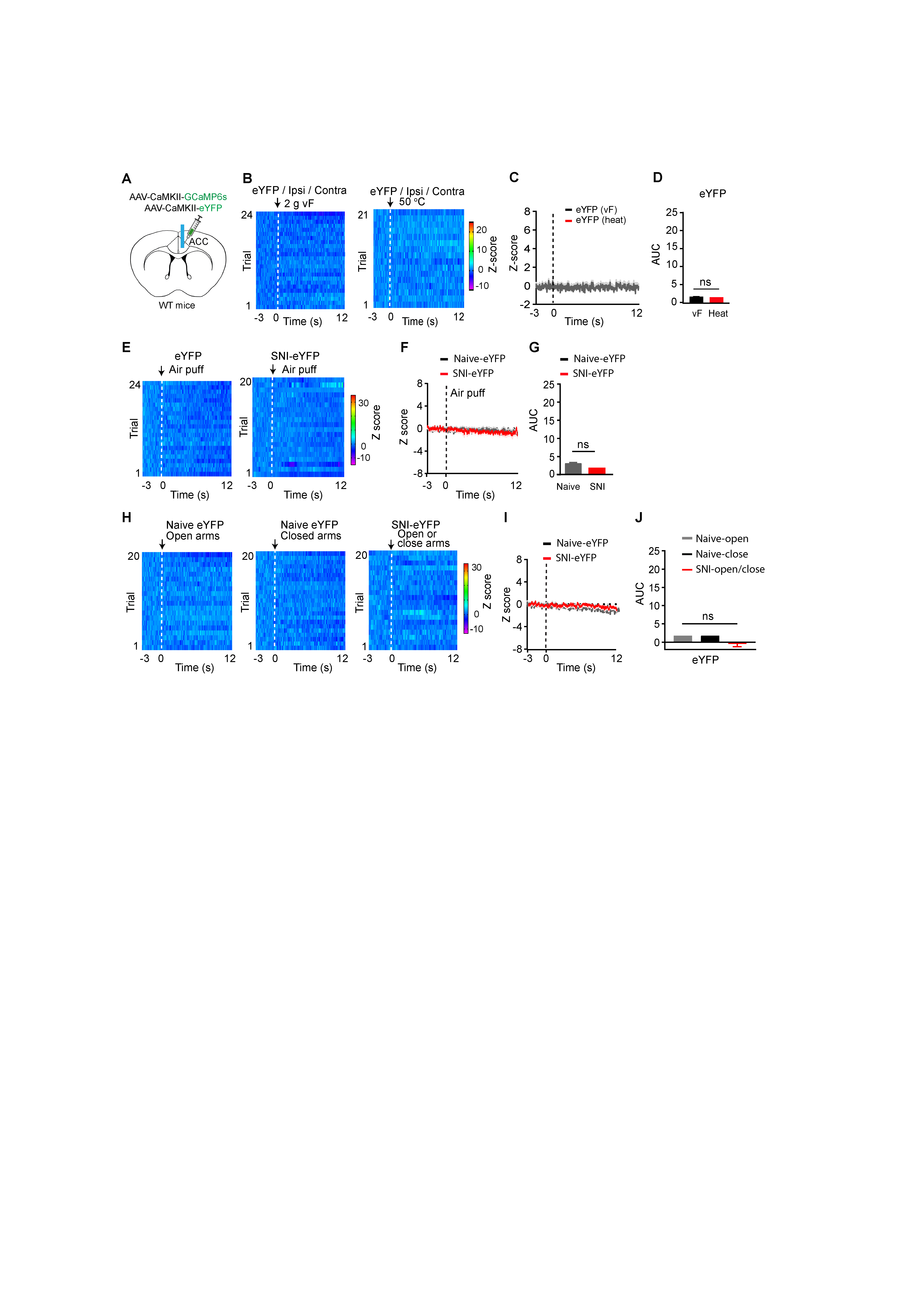

Supplement: S2 Fig — Related to Fig 2. (A) Schematic diagram showing virus (AAV-CaMKII-eYFP) injection and fiber implantation into the ACC for fiberphotometry. (B–D) Heat maps (B), average traces (C), and quantification (D, t = 0.67, P = 0.51) of eYFP signal in the ACC of naïve mice in response to von Frey filament (vF) and thermal stimulation of hind paws. (E–G) Heat maps (E), average traces (F), and quantification (G, t = 0.31, P = 0.67) of eYFP signal in the ACC of naïve or SNI mice expressing eYFP in the ACC in response to 1 s air puff on the face. (H–J) Heat maps (H), average traces (I), and quantification (J, F(2, 57) = 2.69, P = 0.076) of eYFP signal in the ACC of naïve or SNI mice expressing eYFP in the ACC during exploration of the open or closed arms in the elevated plus maze. Two-tailed t test for panels (D, G, n = 5 mice.); one-way ANOVA with Tukey’s post hoc analysis for (J, n = 5 mice in control and SNI groups). ns, not significant. Source data can be found in the second worksheet of S2 Data. (TIFF) [file pbio.3002518.s002.tiff]

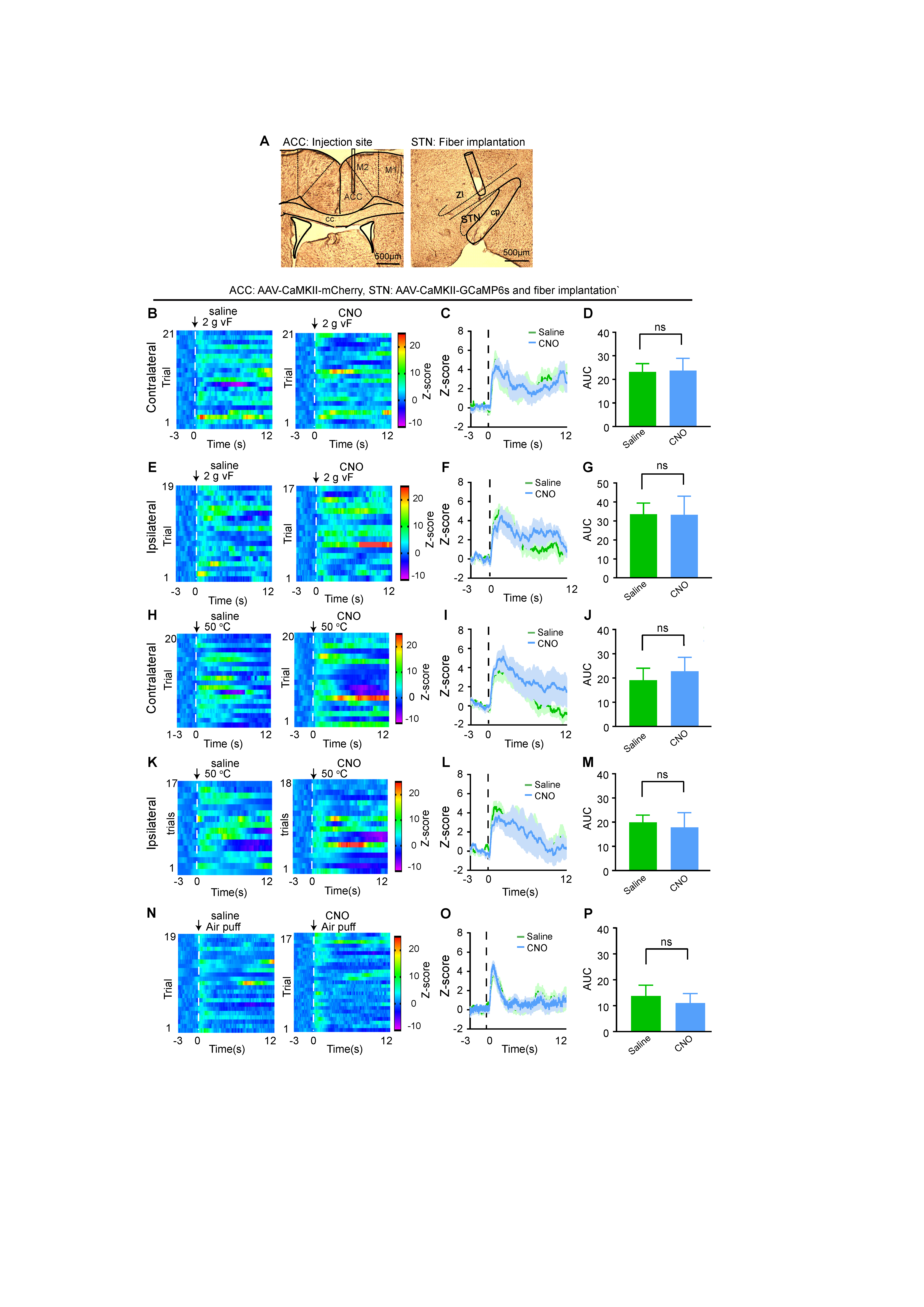

Supplement: S3 Fig — Related to Fig 3. (A) Representative images showing virus injection (AAV-CaMKII-mCherry into the ACC and AAV-CaMKII-GCaMP6s into the STN) and fiber implantation into the STN. (B–M) Heat maps (B, E, H, and K), averaged traces (C, F, I, and L), and quantification (D, G, J, and M) of GCaMP6s signal in the STN of mice receiving von Frey (2 g vF) or 50°C thermal stimulation of hind paws after intraperitoneal injection of either saline or CNO (3 mg/kg). (D) t = 0.073, P = 0.94. (G) t = 0.033, P = 0.97. (J) t = 0.49, P = 0.63. (M) t = 0.30, P = 0.77. (N–P) Heat maps (N), average traces (O), and quantification (P, t = 0.50, P = 0.62) of GCaMP6s signal in the STN of mice exposed to 1 s air puff to the face after intraperitoneal injection of saline or CNO. CNO or the same volume of saline was applied 45 min prior to GCaMP6s signal recording. Dashed lines in panels indicate application of stimuli. Two-tailed t test for (D, G, J, M, and P, n = 5 mice.). ns: not significant. Source data can be found in the third worksheet of S2 Data. (TIFF) [file pbio.3002518.s003.tiff]

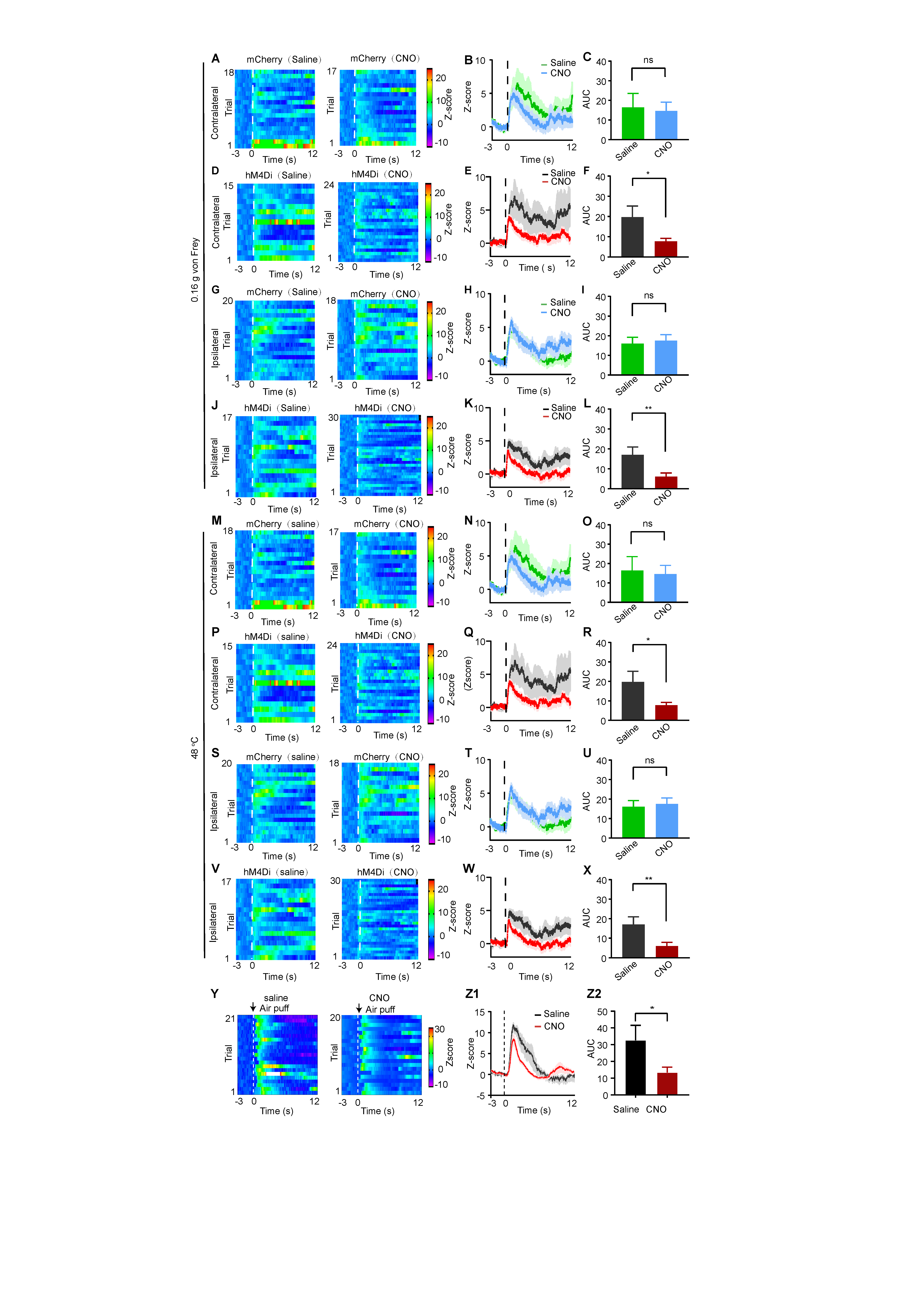

Supplement: S4 Fig — Related to Fig 3. (A–X) Heat maps (A, D, G, J, M, P, S, and V), average traces (B, E, H, K, N, Q, T, and W), and quantification (C, F, I, L, O, R, U, and X) of GCaMP6s signal in the STN of mice with mCherry or hM4Di expression in the ACC in SNI mice. All mice received von Frey (0.16 g) or thermal (48°C) stimulation of hind paws after intraperitoneal injection of saline or CNO (3 mg/kg). (C) t = 0.21, P = 0.84. (F) t = 2.69, P = 0.01. (I) t = 0.33, P = 0.74. (L) t = 2.82, P = 0.0072. (O) t = 1.24, P = 0.22. (R) t = 3.16, P = 0.00027. (U) t = 0.45, P = 0.64. (X) t = 2.77, P = 0.008. (Y–Z2) Heat maps (Y), average traces (Z1), and quantification (Z2, t = 2.15, P = 0.03, unpaired two-tailed t test) of GCaMP6s signal in the STN of SNI mice with hM4Di expression in the ACC in response to 1 s air puff onto the faces after intraperitoneal injection of saline or CNO. CNO or the same volume of saline was applied 45 min prior to fiber photometry recording of GCaMP6s signal. Dashed lines in panels indicate application of stimuli. Two-tailed t test for (C, F, I, L, O, R, U, X, and Z2, n = 5 mice). * P < 0.05, ** P < 0.01; ns, not significant. Source data can be found in the fourth worksheet of S2 Data. (TIFF) [file pbio.3002518.s004.tiff]

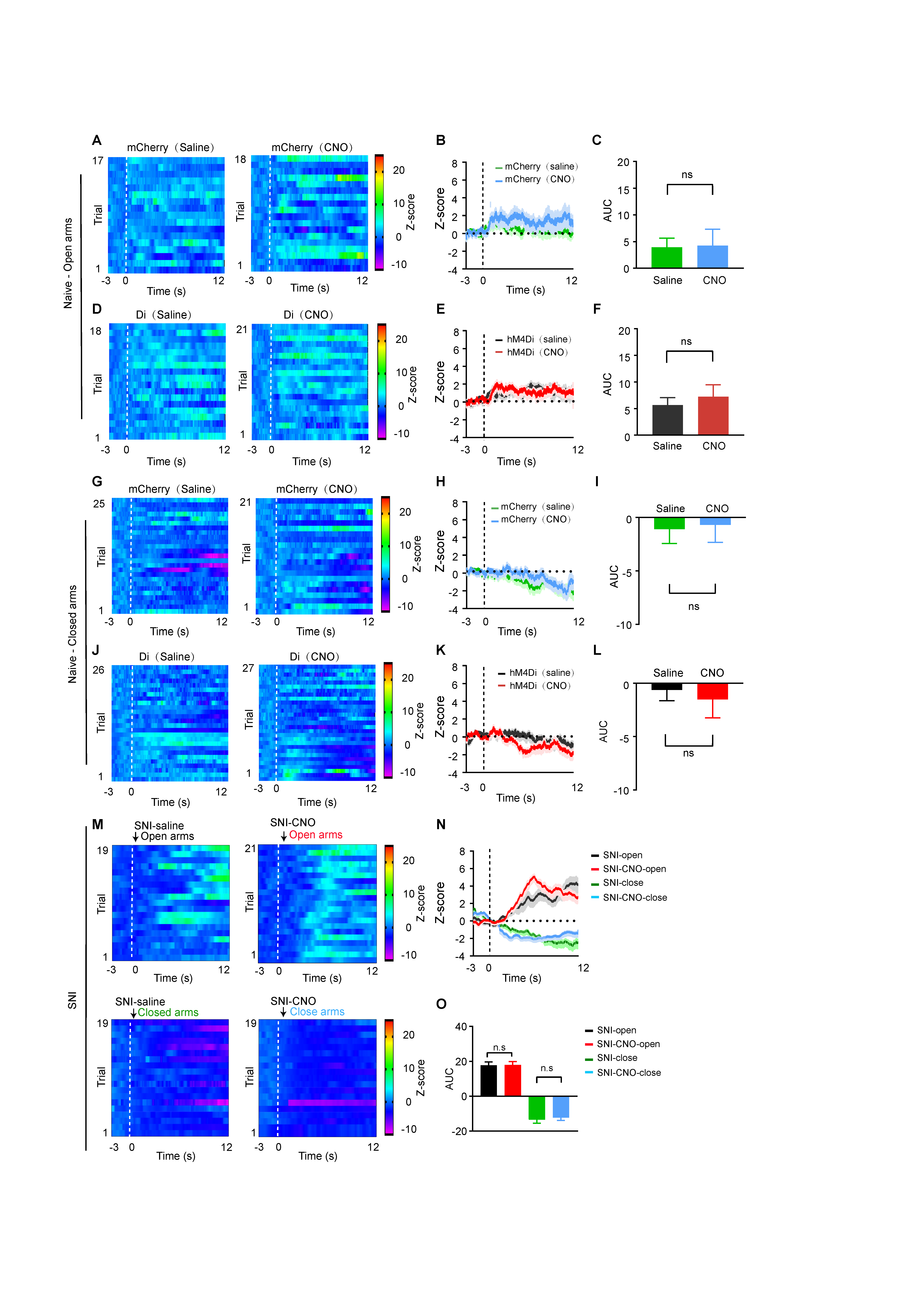

Supplement: S5 Fig — Related to Fig 3. (A–L) Heat maps (A, D, G, and J), average traces (B, E, H, and K), and quantification (C, F, I, and L) of GCaMP6s signal in the STN of naïve mice with mCherry or hM4Di expression in the ACC. All mice explored in the EPM 45 min after intraperitoneal injection of saline or CNO (3 mg/kg). (C) t = 0.081, P = 0.94. (F) t = 0.08, P = 0.57. (I) t = 0.19, P = 0.85. (L) t = 0.42, P = 0.68. (M–O) The hM4Di mice received SNI surgery and fiber photometry recording from the STN were performed 2 weeks later. Changes of GCaMP6s signal in the STN are presented as heat maps (M), average traces (N), and AUC of GCaMP6s signal (O) when the mice entered into the open arms and closed arms 45 min after intraperitoneal administration of saline or CNO. (O) Open arms: t = 0.54, P = 0.59, saline vs. CNO; closed arms: t = 2.37, P = 0.022, Saline vs. CNO. Dashed lines in panels indicate application of stimuli. Two-tailed t test for panels (C, F, I, and L n = 5 mice). One-way ANOVA for (O). ns, not significant. Source data can be found in the fifth worksheet of S2 Data. (TIFF) [file pbio.3002518.s005.tiff]

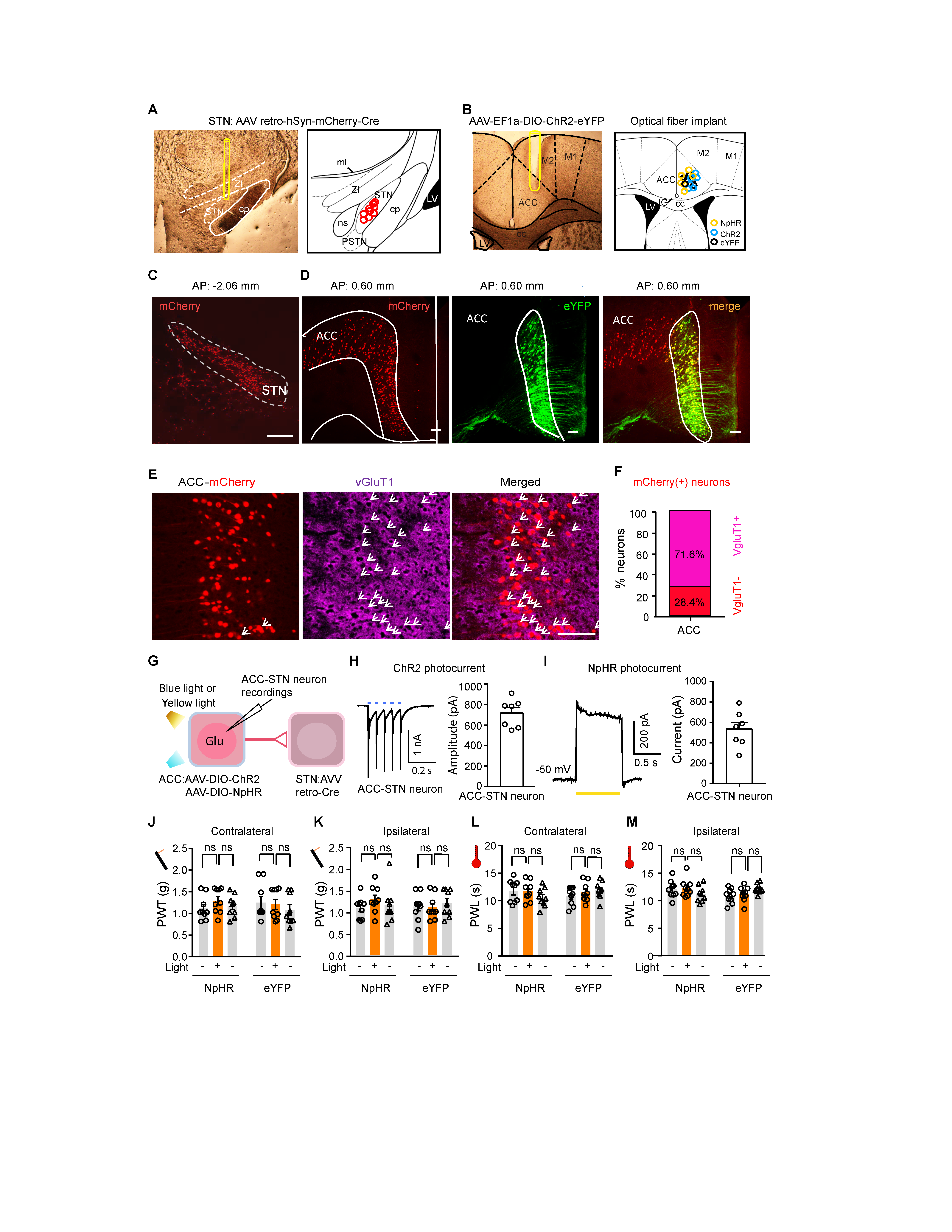

Supplement: S6 Fig — Related to Fig 4. (A and B) Representative image (left) and schematic diagram (right) showing AAV retro-hSyn-mCherry-Cre injection into the STN (A), AAV-EF1α-DIO-ChR2-eYFP injection and optical fiber implantations into the ACC (B) for optogenetic manipulation. (C and D) Example images of mCherry expression in the STN and eYFP expression in mCherry(+) STN projecting ACC neurons. (E and F) Representative images (E) and quantification (F) of mCherry+ neurons (red) labeled with VGluT1-antibody (magenta) (n = 4 mice). (G) Schematic diagram showing recording of ChR2- or NpHR-labeled STN projecting ACC neurons. (H) Representative trace (left) and quantification (right) of corresponding inward currents recorded from ChR2-eYFP-labeled ACC neurons in response to 20 Hz blue light stimulation. n = 7 cells from 3 mice. (I) Representative trace (left) and quantification (right) of corresponding outward currents recorded from NpHR-eYFP-labeled ACC neurons in response to 1 s yellow light stimulation. n = 7 cells from 3 mice. (J–M) Effect of optogenetic silencing of STN-projecting ACC neurons on mechanical PWT (J: Contralateral, F(1, 16) = 0.022, P = 0.88; K: Ipsilateral, F(1, 16) = 0.12, P = 0.73; n = 9 mice) and thermal PWL (L: Contralateral, F(1, 16) = 0.025, P = 0.88, n = 9 mice; M: Ipsilateral, F(1, 16) = 1.09, P = 0.31, n = 9 mice) on both hind paws. Open circles in the right panel of (A) and (B) indicate the locations of virus injection and optical fiber implantations, respectively. *P < 0.05. Two-way repeated measures ANOVA with Tukey’s post hoc analysis for (J–M). Scale bars: 100 μm. Source data can be found in the sixth worksheet of S2 Data. (TIFF) [file pbio.3002518.s006.tiff]

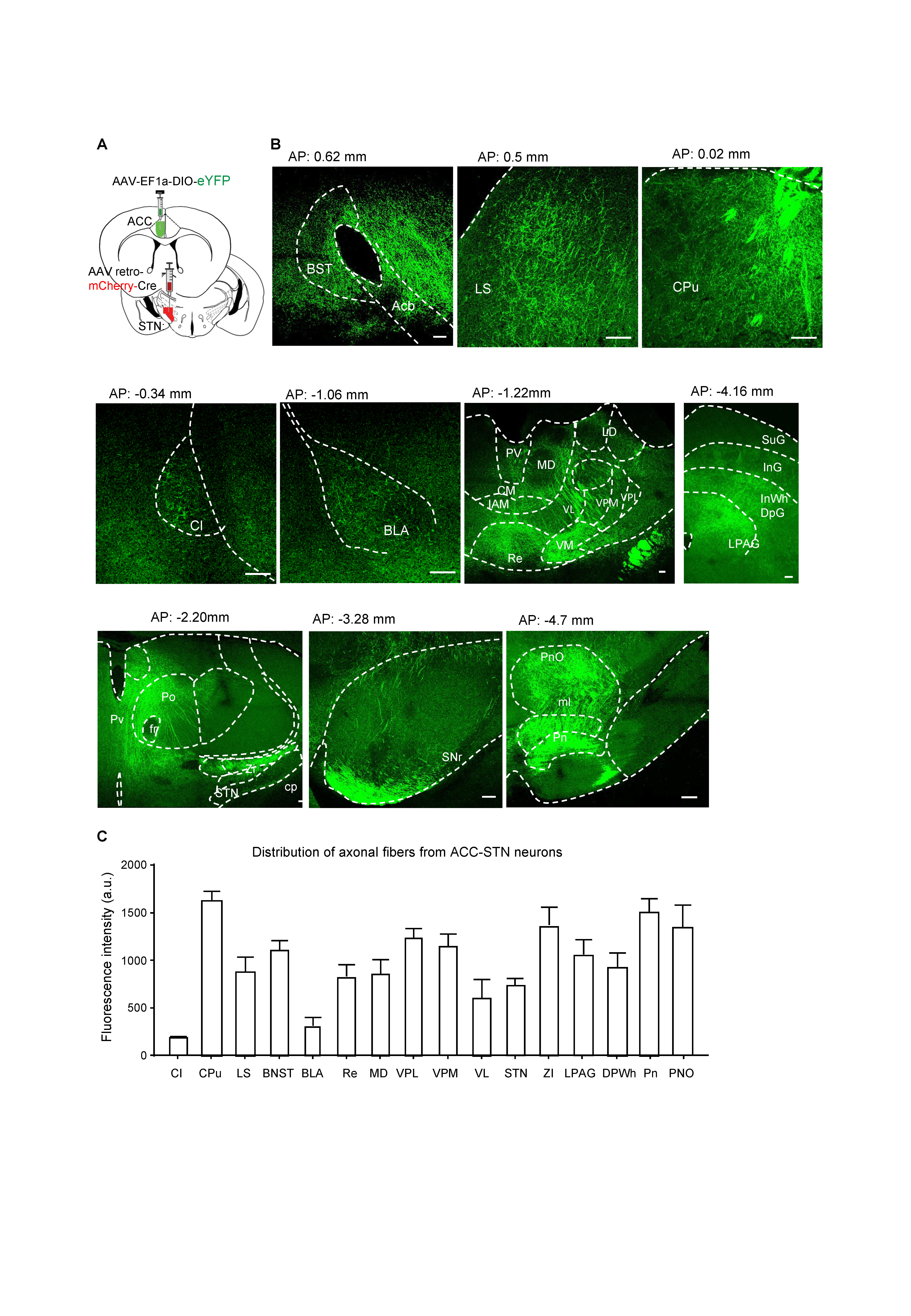

Supplement: S7 Fig — Related to Fig 5. (A) Schematic diagram showing locations of virus injection. AAV retro-hSyn-mCherry-Cre was injected into the STN and AAV-EF1α-DIO-eYFP was injected into the ACC to label ACC-STN neurons and their projecitons. (B and C) Representative images and quantification for distribution of eYFP-labeled axonal fibers in brain regions. n = 4 mice. Acb: nucleus accumbens; BLA: basolateral amygdala; BST: bed nucleus of the stria terminalis; Cl: claustram; CM: centromedial thalamus; cp: cerebral peduncle; CPu: caudate putamen; InWh: Intermediate white layer of the superior colliculus; LPAG: lateral periaqueductal gray; LS: lateral septum; PV: paraventricular thalamic nucleus; MD: mediodorsal thalamus; IMA: intramedullary thalamus; LD: laterodorsal thalamus; ml: medial lemniscus; Pn: Pontine nucleus; PnO: pontine reticular oral part; Po: posterior thalamus; VL: ventrolateral thalamus; VM: ventromedial thalamus; VPM and VPL: ventroposterior thalamus medial and lateral part; Re: reuniens thalamus; SNr: substantial nigra pars reticulata; STN: subthalamic nucleus; SuG, InG and DpG: superficial, intermediate and deep gray layer of the superior colliculus; Zi: zona incerta. Scale bars: 100 μm. Source data can be found in the seventh worksheet of S2 Data. (TIFF) [file pbio.3002518.s007.tiff]

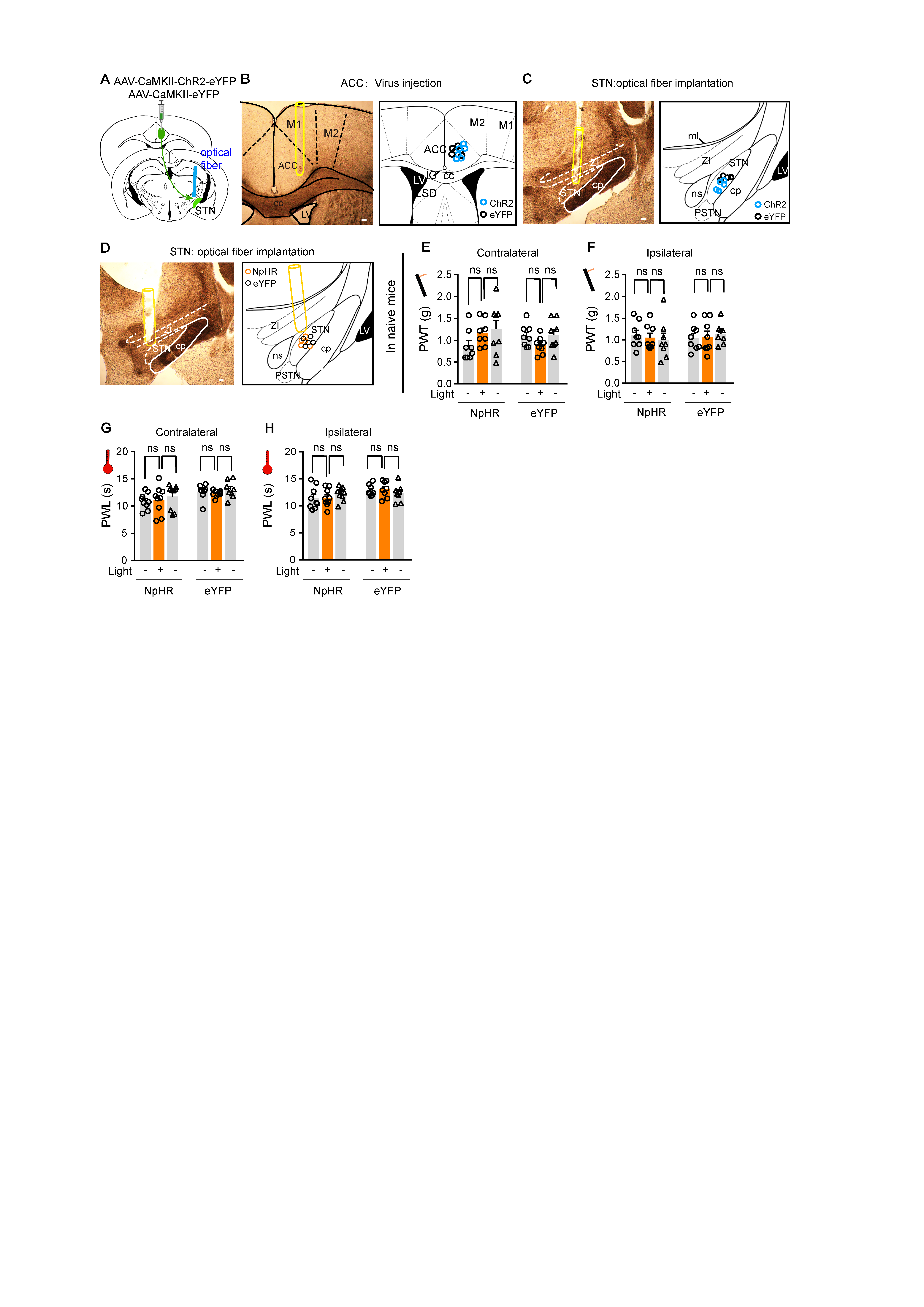

Supplement: S8 Fig — Related to Fig 6. (A–C) Schematic diagram of virus injection (A and B) and optical fiber implantation (C) for optogenetic activation of the ACC-STN projection. Right panels in (B) and (C) summarize locations of virus injections into the ACC and optical fiber implantations into the STN, respectively. (D) A representative image (left) and locations of optical fiber implantations (right) in the STN for optogenetic inhibition of the ACC-STN projection. (E–H) Effect of yellow light illumination (589 nm, continuous, 3 mW) of the ACC-STN projection on mechanical PWT and thermal PWL in naïve mice. (E) F(2, 28) = 2.35, P = 0.11; (F) F(2, 28) = 0.67, P = 0.52; n = 8 mice in each group; (G) F(2, 30) = 0.12, P = 0.89; (H) F(2, 30) = 1.75, P = 0.19. n = 9 NpHR mice, n = 8 eYFP mice. Two-way repeated measures ANOVA with Tukey’s post hoc analysis for (E–H). ns, not significant. Scale bars: 100 μm. Source data can be found in the eighth worksheet of S2 Data. (TIFF) [file pbio.3002518.s008.tiff]

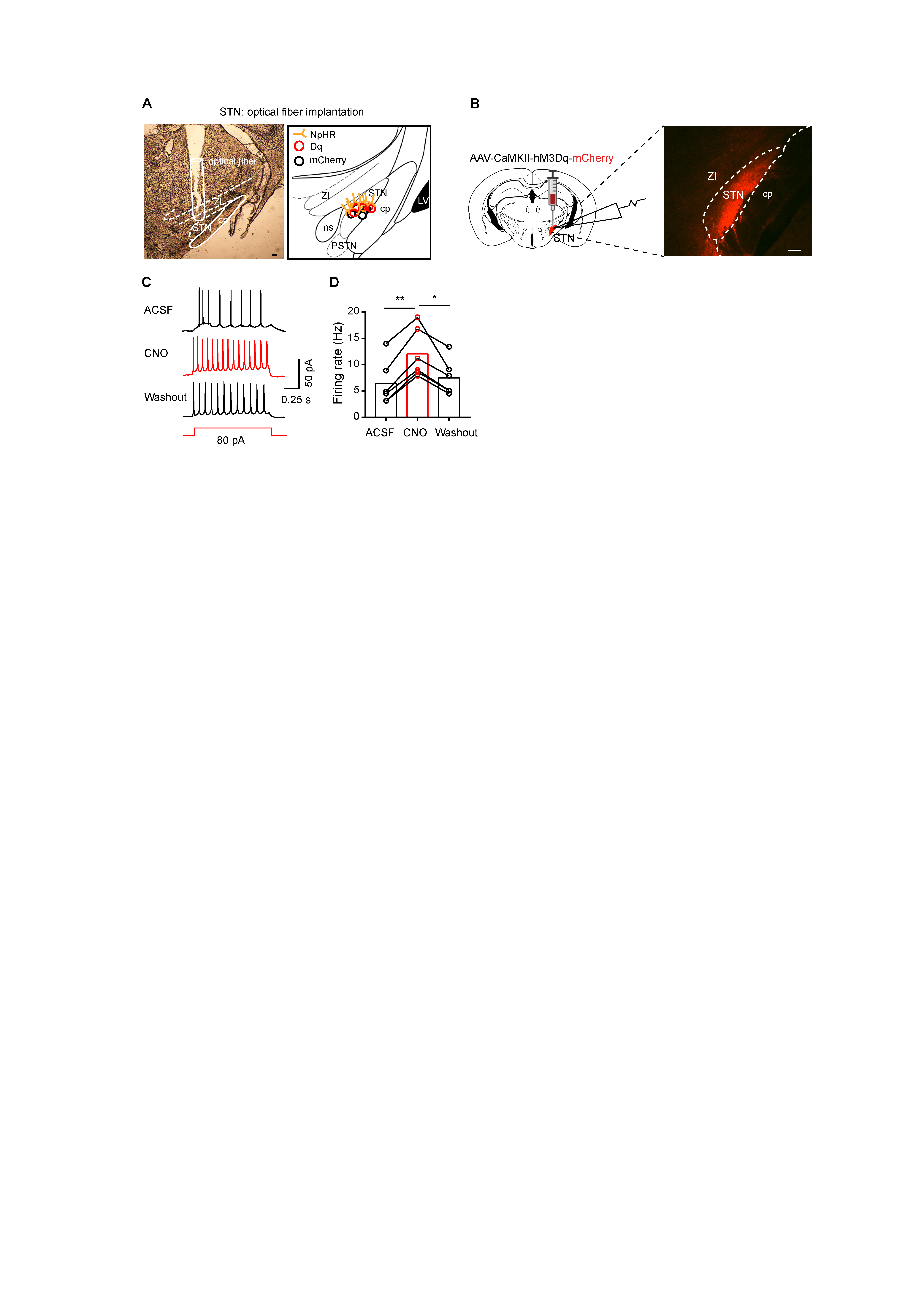

Supplement: S9 Fig — Related to Fig 6. (A) A representative image and summary for the locations of optical fiber implants in the STN for optogenetic inhibition of the ACC-STN projections and virus injection sites for chemogenetic activation of STN neurons. (B) Left: Schematic diagram of virus injections for chemogenetic activation of STN neurons; right: representative image of hM3Dq-mCherry-labeled STN neurons. (C and D) Representative traces and quantification of evoked action potentials before, during (5 min), and 10 min after termination of CNO (3 μM) perfusion. Firing rate: F(1.2, 6.001) = 17.17, P = 0.0051. n = 6 neurons. One-way repeated measures ANOVA with Tukey’s post hoc analysis for (D). * P < 0.05; P < 0.01. Scale bars: 100 μm. Source data can be found in the ninth worksheet of S2 Data. (TIFF) [file pbio.3002518.s009.tiff]

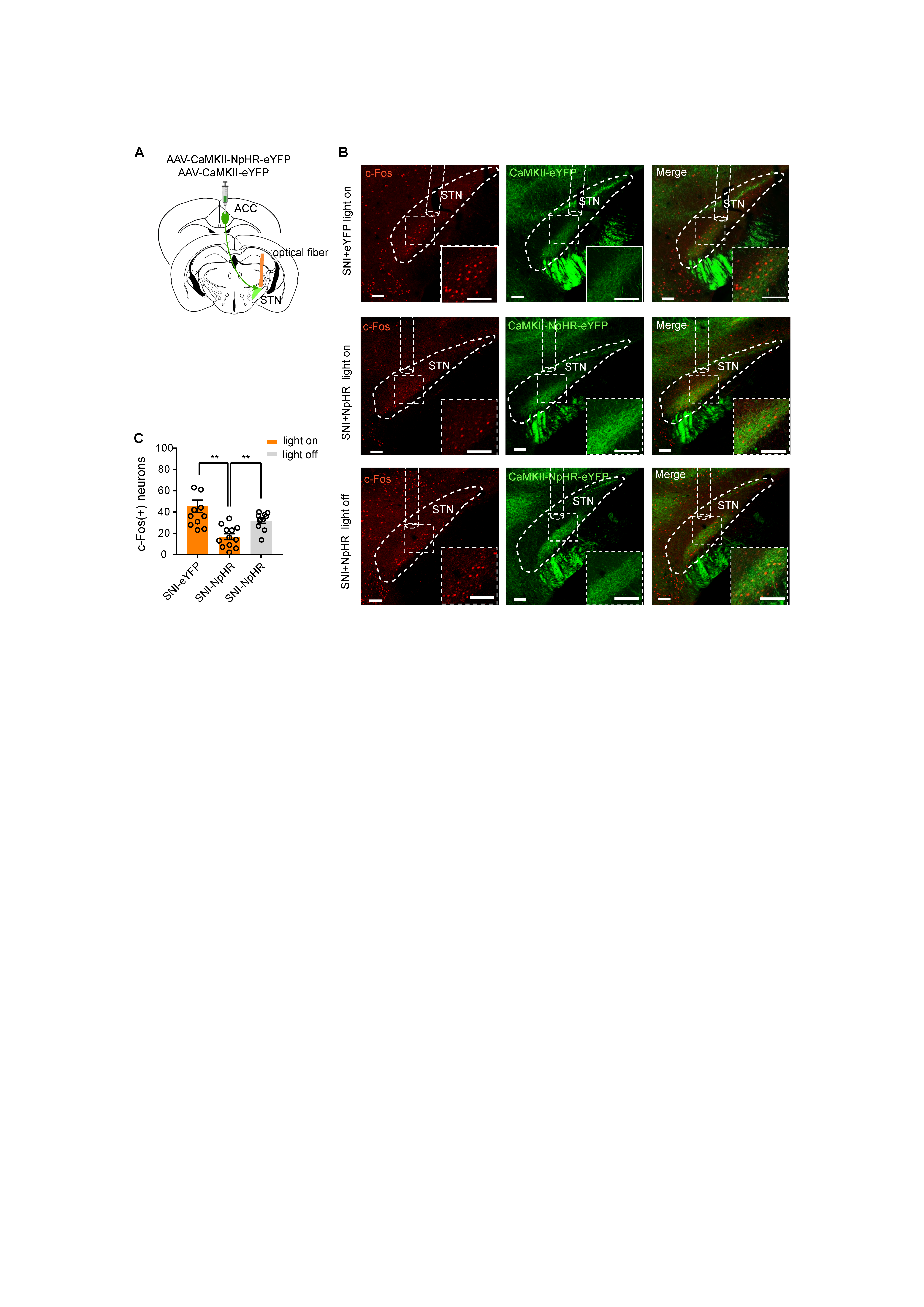

Supplement: S10 Fig — Related to Fig 6. (A) Schematic diagram of virus injection and fiber implantation for optogenetic silencing of the ACC-STN projection. Yellow light was delivered into the STN for 20 min (589 nm, continuous, 3 mW, 2 min episodes with 30 s intervals) in NpHR- and eYFP-expressing mice 4 weeks after SNI surgery. The mice were sacrificed 1 h after yellow light illumination for c-Fos-staining. (B and C) Representative images (B) and quantification (C) of c-Fos(+) STN neurons. (C) F(2, 32) = 12.54, P < 0.0001. One-way ANOVA with Tukey’s post hoc analysis. ** P < 0.01, n = 12 slices from 4 mice in each group. Scale bars: 100 μm. Source data can be found in the tenth worksheet of S2 Data. (TIFF) [file pbio.3002518.s010.tiff]
