# Supplementary material for: The anterior cingulate cortex controls the hyperactivity in subthalamic neurons in male mice with comorbid chronic pain and depression
Source: PLoS Biol. 2024 Feb 22;22(2):e3002518. doi: 10.1371/journal.pbio.3002518 (PMC10883538; doi:10.1371/journal.pbio.3002518)
Supplement: S1 Table — Related to Figs 3 and S4. Source data can be found in S3 Data. (DOCX) [file pbio.3002518.s011.docx]

|  | Contralateral  von Frey | Ipsilateral  von Frey | Contralateral  heat | Ipsilateral  heat |
| --- | --- | --- | --- | --- |
| Naive | 23.25±3.48  (n = 16) (2 g) | 33.57 ± 5.86  (n = 13) (2 g) | 19.13 ± 4.92  (n = 19) (50 ℃) | 19.88 ± 3.06  (n = 16) (50 ℃) |
| SNI | 16.4±7.21  (n = 17) (0.16 g) | 19.75 ± 3.46  (n = 15) (0.16 g) | 18.32 ± 3.89  (n = 21) (48 ℃) | 13.18 ± 4.15  (n = 22) (48 ℃) |
| Two-tailed unpaired t- test | t = 0.84  P = 0.41 | t = 2.1  P = 0.05 | t = 0.13  P = 0.89 | t = 0.083  P = 0.93 |

**S1 Table**

**Changes of GCaMP6s signal in the STN in response to pain stimulation**

Source data are in supporting information (S3_Data.xlsx).
