# Supplementary material for: The anterior cingulate cortex controls the hyperactivity in subthalamic neurons in male mice with comorbid chronic pain and depression
Source: PLoS Biol. 2024 Feb 22;22(2):e3002518. doi: 10.1371/journal.pbio.3002518 (PMC10883538; doi:10.1371/journal.pbio.3002518)
Supplement: S2 Table — Related to Figs 3, S4, and S5. Source data can be found in S3 Data. (DOCX) [file pbio.3002518.s012.docx]

**S2 Table**

**Changes of GCaMP6s signal in the STN in response to air puff and during exploration in the elevated plus maze**

|  | Air puff | EPM open arms | EPM closed arms |
| --- | --- | --- | --- |
| Naive | 13.81± 4.15  (n = 13) | 5.68 ± 1.4  (n = 18) | -1.88 ± 1.13  (n = 23) |
| SNI | 22.09± 3.45  (n = 17) | 17.70 ± 1.96  (n = 21) | -13.45 ± 2.03  (n = 19) |
| Two-tailed unpaired t- test | t = 1.55  P = 0.13 | t = 4.85  P < 0.0001 | t = 5.21  P < 0.0001 |

Source data are in supporting information (S3_Data.xlsx).
